# Ferroptosis: An Energetic Villain of Age-Related Macular Degeneration

**DOI:** 10.3390/biomedicines13040986

**Published:** 2025-04-17

**Authors:** Na Zhao, Siyu Li, Hao Wu, Dong Wei, Ning Pu, Kexin Wang, Yashuang Liu, Ye Tao, Zongming Song

**Affiliations:** 1 Henan Eye Institute, Henan Eye Hospital, People’s Hospital of Zhengzhou University, Henan University School of Medicine, Henan Provincial People’s Hospital, Zhengzhou 450003, China; 13949985339@163.com (N.Z.); wkx20000121@gs.zzu.edu.cn (K.W.); l15136816206@gs.zzu.edu.cn (Y.L.); 2 College of Medicine, Zhengzhou University, Zhengzhou 450001, China; lsiyu@gs.zzu.edu.cn (S.L.); q1w1e1a1s1d@stu.zzu.edu.cn (H.W.); 202047000423@stu.zzu.edu.cn (D.W.); 202051081418@syu.zzu.edu.cn (N.P.)

**Keywords:** age-related macular degeneration, iron, lipid peroxidation, ferroptosis

## Abstract

Iron homeostasis plays an important role in maintaining cellular homeostasis; however, excessive iron can promote the production of reactive oxygen species (ROS). Ferroptosis is iron-dependent programmed cell death that is characterized by excessive iron accumulation, elevated lipid peroxides, and the overproduction of ROS. The maintenance of iron homeostasis is contingent upon the activity of the transferrin receptor (TfR), ferritin (Ft), and ferroportin (FPn). In the retina, iron accumulation and lipid peroxidation can contribute to the development of age-related macular degeneration (AMD). This phenomenon can be explained by the occurrence of the Fenton reaction, in which the interaction between divalent iron and hydrogen peroxide leads to the generation of highly reactive hydroxyl radicals. The hydroxyl radicals exhibit a propensity to attack proteins, lipids, nucleic acids, and carbohydrates, thereby instigating oxidative damage and promoting lipid peroxidation. Ultimately, these processes culminate in cell death and retinal degeneration. In this context, a comprehensive understanding of the exact mechanisms underlying ferroptosis may hold significant importance for developing therapeutic interventions. This review summarizes recent findings on iron metabolism, cellular ferroptosis, and lipid metabolism in the aging retina. We also introduce developments in the therapeutic strategies using iron chelating agents. Further refinements of these knowledges would deepen our comprehension of the pathophysiology of AMD and advance the clinical management of degenerative retinopathy. A comprehensive search strategy was employed to identify relevant studies on the role of ferroptosis in AMD. We performed systematic searches of the PubMed and Web of Science electronic databases from inception to the current date. The keywords used in the search included “ferroptosis”, “AMD”, “age-related macular degeneration”, “iron metabolism”, “oxidative stress”, and “ferroptosis pathways”. Peer-reviewed articles, including original research, reviews, meta-analyses, and clinical studies, were included in this paper, with a focus on the molecular mechanisms of ferroptosis in AMDs. Studies not directly related to ferroptosis, iron metabolism, or oxidative stress in the context of AMD were excluded. Furthermore, articles that lacked sufficient data or were not peer-reviewed (e.g., conference abstracts, editorials, or opinion pieces) were not considered.

## 1. Introduction

Age-related macular degeneration (AMD) is a progressive retinal disease that initially affects the macula and gradually extends to the neighboring area of the posterior retina. It acts as the main cause of irreversible visual loss in the population over 65 years of age [1]. Retinal pigment epithelium (RPE) cells are arranged as single-layer polygonal cells with sharp ends (mostly hexagonal) in the eyes of healthy adults. Age greatly reduces the number of hexagonal cells [2]. The RPE performs numerous critical functions for the choroid and photoreceptors, including phagocytosis of photoreceptor outer segments, light absorption, processing of retinoids for phototransduction (visual cycle), maintenance of the blood–retina barrier, secretion of lipoprotein particles, growth factors, and cytokines [3]. As RPE cells undergo aging, they have the potential to accumulate detrimental substances such as metabolites, aging cell debris, and lipid peroxides. The accumulation of these substances can impede cell function, disrupt cell metabolism, and hinder the delivery of essential nutrients. Moreover, the antioxidant defense system of RPE cells may weaken with age, resulting in heightened oxidative stress [4]. This oxidative stress triggers an inflammatory response and leads to cellular damage and sustained inflammation [5]. Additionally, RPE cells play a crucial role in maintaining the well-being of visual cells. However, as RPE cells age, their ability to effectively regulate the supply of oxygen and nutrients to visual cells may diminish. Gradually, they lose their capacity to fulfill their regulatory function for retinal photoreceptors, culminating in the demise of photoreceptors [3]. This process consequently expedites the progression of AMD. Early-stage AMD is characterized by the formation of vitreous warts and the abnormal morphology of the RPE. Alterations in the skeleton of RPE cells can result in changes in the shape of normal regular polygons, as well as symptoms of RPE cell death and abnormal pigmentation. As the disease progresses over time, the RPE cells expand and fuse, resulting in focal division, fragmentation, and the appearance of intracellular stress fibers [6]. Late-stage AMD causes permanent vision impairment that seriously affects the life quality of patients, and is divided into dry AMD (known as atrophic or non-exudative) and wet AMD types (also known as neovascular or exudative). Dry AMD is accompanied by the formation of vitreous warts, which eventually leads to the apoptosis of RPE cells [7]. Wet AMD is characterized by the formation of neovascularization, which leads to liquid leakage, retinal edema, the proliferation of glia cells, and the formation of fiber scars. In particular, severe AMD induces irreversible macular damage [8]. The macula situated in the central area of the retina is composed of a significant population of light-sensitive nerve cells, particularly cone cells. These specialized cells are essential for perceiving intricate visual details and recognizing colors [9]. In AMD pathology, cone photoreceptors degeneration leads to macular thinning and central visual blurring [10]. Therefore, anti-vascular endothelial growth factor (anti-VEGF) drugs have certain curative effects on wet AMD by inhibiting the formation of neovascularization. However, there is no effective treatment for dry AMD at this time [11]. This dilemma should be ascribed to the complex etiologic backgrounds of AMD. Age, genetic mutations, living habits, and environmental factors collectively contribute to the pathogenesis of AMD [12]. Therefore, characterizing the exact pathological mechanism underlying AMD is essential for developing future therapies.

Iron is a metal element that is necessary during biological growth and development. However, excessive iron can induce functional abnormalities and pathological damages. Accumulating evidence shows that ferroptosis, a form of iron-dependent programmed cell death (PCD), is implicated in the pathology of degenerative retinopathy [13,14]. The typical characteristics of ferroptosis-mediated PCD include iron overload, reduced membrane density, the recession of the mitochondrial crest, and the rupture of the outer membrane [15]. In terms of cell composition, lipid peroxide and ROS increase. As a consequence of the accumulation of free iron and the reactive oxygen species (ROS), the internal environment of the cell undergoes gradual changes, which can disrupt the ion balance. This disturbance extends to the acid–base balance of cells, causing fluctuations in PH both inside and outside cells. Consequently, the normal metabolism and functioning of cells are compromised, impacting their regular physiological processes. Moreover, the accumulation of free iron and ROS can lead to alterations in cell membrane. This can manifest as abnormal activity in ion channels and carrier proteins in the membrane, resulting in disturbed ion balance and the impaired transport and excretion of substances outside the cell. The ferroptosis process is mainly modulated by the action of ferrous iron (Fe^2+^) or lipoxygenase (LOX). The high expression of saturated fatty acids on the cell membrane is catalyzed and then lipid peroxide occurs [16]. Fe^2+^ is the active form of iron ion, and under normal physiological conditions, most of the iron exists in the ferric state (Fe^3+^). Esterases, such as hepcidin (Hepc) and ceruloplasmin (Cp), play a role in converting Fe^2+^ to Fe^3+^, thereby limiting the presence of active iron [17]. However, when there is an excess accumulation of free iron in cells, this triggers the production of oxygen radicals and promotes the generation of lipid peroxides [18]. This accumulation impairs the cell’s ability to effectively eliminate lipid peroxidation and maintain lipid metabolism balance. Consequently, the lipid peroxidation process accumulates and the negative feedback mechanism is disrupted, ultimately leading to cell death. To regulate the uptake of iron in the cell, the synergistic effects of Fe^2+^ and esterases come into play. They inhibit the expression or reduce the activity of transferrin receptor 1 (TfR1), which helps reduce the uptake of iron. This mechanism prevents iron from causing oxidative stress and intracellular damage.

In parallel with the oxidative damage, ferroptosis is also characterized by deficits in the antioxidant system, such as the reduced expressions of glutathione (GSH) and glutathione peroxidase 4 (GPX4) [19]. Cysteine is recognized as the rate-limiting precursor for GSH synthesis [20]. It is absorbed as cystine by the system Xc- (also named cystine/glutamate anti-transporter). The system Xc- is composed of the heavy chain subunit SLC3A2 and the light chain subunit SLC7A11 [21]. The primary role of the system Xc- is to transport cystine into cells while simultaneously exporting glutamate in a 1:1 ratio. Generally, the system Xc- facilitates the uptake of cystine, which is rapidly reduced to cysteine inside the cells. Within the cellular environment, cysteine and glutamate combine through the action of glutamylcysteine ligase (GCL) to form γ-glutamylcysteine (γGC). Subsequently, the addition of glycine catalyzed by glutathione synthase results in the production of the tripeptide GSH [22]. GSH, a non-enzymatic antioxidant, plays a crucial role in protecting cells from oxidative stress. It is found in high concentrations in the neuroretina and RPE [23]. The antioxidant properties of GSH are attributed to its capacity to neutralize ROS and serve as a vital cofactor for enzymes such as GSH S-transferase and peroxidase [24]. Intracellular levels of cysteine and GSH play a pivotal role in regulating GPX4 activity. GSH, serving as a major co-factor for GPX4, functions as both an electron donor and acceptor through the interconversion between its reduced form (G-SH) and oxidized form (GS-SG). This dynamic redox process is vital for mitigating oxidative stress [25]. GPX4 is responsible for catalyzing the reduction of phospholipid hydroperoxides (PLOOH) to their corresponding hydroxyl derivatives [26]. In the catalytic cycle of GPX4, the main active group, -SeH, is oxidized to selenic acid (-SeOH) by PLOOH. However, G-SH can reduce -SeOH and effectively reactivate GPX4, involving the release of GS-SG, which helps prevent GPX4 inactivation [27]. Overall, the interplay between intracellular cysteine, GSH, and GPX4 is crucial for maintaining the antioxidant defense system [28]. System Xc- activity ensures the maintenance of optimal GSH levels, which directly participate in neutralizing oxidative stress as a coenzyme GSH for GPX4. GPX4, in turn, acts to counter ferroptosis by reducing the buildup of lipid peroxides. These interconnected mechanisms synergistically provide the cell with an effective defense system, safeguarding it against the detrimental effects of ferroptosis. During apoptosis, the integrity of the plasma membrane is preserved through the hydrolysis and digestion of the cellular component. However, ferroptosis can cause the plasma membrane rupture, and the subsequent release of cell contents, thereby initiating the inflammatory response [29]. Typically, the system Xc- -GSH- GPX4 aix acts as the main mechanism for preventing ferroptosis [30].

In recent decades, several forms of regulated cell death have been shown to contribite to the pathogenesis of AMD, including apoptosis, autophagy, and focal death, etc., [4,31,32]. Among them, ferroptosis is a unique mode of cell death driven by iron-dependent phospholipid peroxidation. Abnormal iron accumulation in the retina is closely implicated in the pathology of AMD [33,34]. Generally, ferroptosis is regulated by a variety of cellular metabolic events, such as redox reactions, iron handling, mitochondrial activity, and the metabolism of amino acids, lipids, and sugars [35]. Accumulating evidence suggests that the oxidative stress and lipid peroxidation can contribute to AMD progression [5]. High concentrations of polyunsaturated fatty acids (PUFAs) in the outer segment of photoreceptors act as the main source of intracellular ROS, which make the retina particularly vulnerable to oxidative stress-mediated damages [5]. Oxidative stress arises when cells or tissue generate excessive ROS, including superoxide anions, hydrogen peroxide, hydroxyl radicals, and others [36]. RPE phagocytoses the POS shed from photoreceptors that contain photosensitive groups, various oxidants, and unsaturated fatty acids, which leads to the increased production of ROS. In addition, aging is another risk factor that induces oxidative-stressed damage in the RPE. With aging, the number of dysfunctional mitochondria increases in the RPE, leading to a significant increase in ROS generation [37]. cGAS-STING activation has been detected in oxidative stress-induced retina degeneration, accompanied with cytosolic leakage of damaged DNA in photoreceptors. Pharmaceutical and genetic approaches suggest that STING promotes retina inflammation and degeneration upon oxidative damage. Drug screening reveals that BRD4 inhibitor JQ1 reduces cGAS-STING activation, inflammation, and photoreceptor degeneration in the injured retina [38]. Aging further involves the accumulation of oxidative stress, and marked increases in the expression of oxidized proteins or lipids can be detected in the aging retina. Senescent cells may secrete pro-inflammatory cytokines and chemokines, such as IL-6, IL-8, TNF, IL-1α, IL-1β, MCP-1, MCP-2, CX3CL1, IGF–IGFR, and colony-stimulating factors (GCSF and GMCSF). These mediators further stimulate microglia or macrophages and the tissue complement system. These stressed cells may accelerate the progress of AMD [39]. Furthermore, the regulation of the mitochondrial function and antioxidant stress response by non-coding RNAs (ncRNAs), newly emerged epigenetic factors, has been discussed. Downregulation of miR-181a and b would be able to protect retinal neurons from death, as these miRNAs directly target the genes of nuclear respiratory factor 1 (NRF1), COX11, coenzyme Q (ubiquinone) binding protein COQ10 homolog B (COQ10B), and peroxiredoxin 3 (PRDX3), all of which are important in mitochondrial biogenesis and functioning [40]. In the presence of oxidative stress, free iron can act as a catalyst, exacerbating the generation of harmful free radicals. Iron reacts with hydrogen peroxide (H_2_O_2_), leading to the formation of highly reactive hydroxyl radicals. These radicals inflict damage on various cellular components, such as proteins, lipids, and nucleic acids. Consequently, cell membranes rupture, mitochondrial function becomes impaired, DNA sustains damage, and so forth. This chain of events ultimately leads to the occurrence of ferroptosis [41]. Therefore, ferroptosis provides a new therapeutic target for AMD. In this review, we summarize recent findings on the contributory role of ferroptosis to AMD pathology, and focus on its therapeutic potentials in developing treatment.

## 2. Pathological Features of AMD

**Figure 1:**
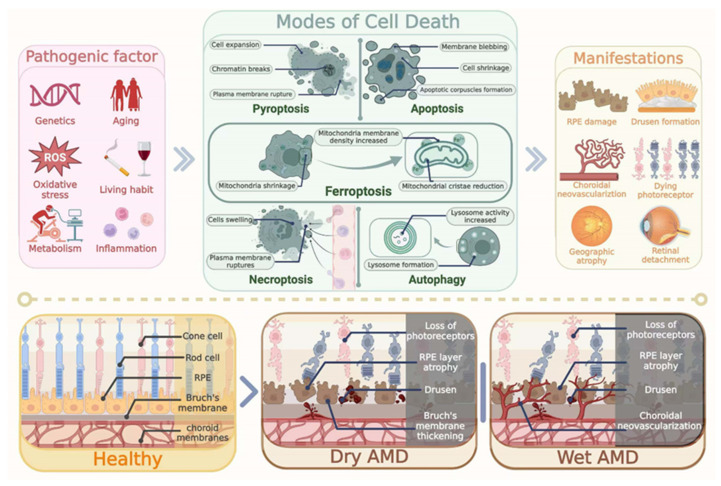
Types of age-related macular degeneration, pathogenic factors, and styles of cell death. Genetic mutations, aging, oxidative stress, living habits, metabolism, and inflammation are contributed to the pathogenesis of AMD. The main pathological feature of AMD is characterized by RPE damage, the presence of drusen, geographic atrophy, and dying photoreceptors, along with complications like choroidal neovascularization and retinal detachment. Nevertheless, AMD is a multifaceted disorder, including various forms of cell death. Pyroptosis, apoptosis, ferroptosis, necroptosis, and autophagy contribute to retinal cell demise. AMD, age-related macular degeneration.

The main pathological feature of dry AMD is drusen formations. Drusen is a yellowish deposit between the Bruch membrane and the RPE cell layer. Drusen formation should be ascribed to the inability of RPE to phagocytize and digest the shredded outer segment of photoreceptors [42]. Under physiological conditions, healthy RPE cells have a strong phagocytosis ability to remove the corpuscles derived from the shredded outer segment. In AMD, the phagocytic ability of an RPE cell is impaired and the corpuscles cannot be cleared in time. These corpuscles finally deposit in the Bruch membrane and form vitreous warts and drusen [43]. As the RPE density in the macula is remarkably higher compared with the rest of the region of the retina, the macula is the most vulnerable to abnormalities in RPE function. These disturbances promote the development of dry AMD and lead to the loss of central vision [44,45]. With the progression of AMD, the accumulation of drusen can trigger inflammation [46]. Drusen contains various cytokines and inflammatory mediators, including tumor necrosis factor-alpha (TNF-α), interleukin-1 (IL-1), interleukin-6 (IL-6), prostaglandins, oxidative stressors, and nitric oxide. These cytokines can activate microglia, macrophages, and other IL-6-expressing cells [47]. The activated neuroglia and RPE cells can secrete VEGF, which promotes the growth of new retinal vessels. Neovascularization is a pathological hallmark of wet AMD, which eventually leads to macular detachment [48]. Notably, abnormal iron accumulation has been found in aqueous humor and retinal tissue of AMD patients, highlighting that iron plays a critical role in the pathogenesis of AMD (Figure 1) [49].

## 3. Regulation of Iron in Retina

### 3.1. Iron Homeostasis in Normal Retina

**Figure 2:**
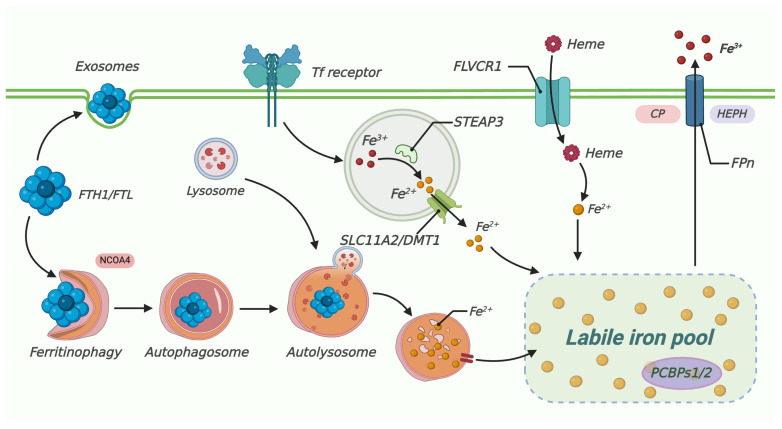
Schematic diagram of signaling pathways in iron homeostasis. Fe^3+^ dissociates from the Tf-TfR complex, which is followed by reduction of Fe^3+^ to Fe^2+^ catalyzed by STEAP3 and then transported across the endosomal membrane into the cytoplasm by the ferrous iron transporter DMT1. Excess Fe^2+^ enters the labile iron pool as the source of active-redox iron. NCOA4 facilitates the delivery of the iron-storage protein Ft to the lysosomes, where Ft undergoes degradation. The released Fe^2+^ from degraded Ft contributes to the labile iron pool in the cytoplasm. Some common iron-chelating agents include deferoxamine, deferiprone, deferasirox, salicylaldehyde isonicotinoyl hydrazine, VK28, M30, etc. Tf, transferrin; STEAP3, six-transmembrane epithelial antigen of prostate 3; DMT1, divalent metal transporter 1; NCOA4, nuclear receptor coactive 4.

Retina is a photosensory tissue with complicated architectures and complex physical activities [50]. Iron acts as an auxiliary factor for the phototransduction enzyme, which is exclusively expressed in photoreceptors and RPE cells. Iron plays a crucial role in the optical cascade of vision. RPE65 is an iron-dependent isomer hydrolase in the visual cycle [51]. The RPE65 protein is located in RPE, which converts 11-cis-retinaldehyde into all-trans retinaldehyde, and is involved in vitamin A-like circulation. In addition, iron is necessary for the synthesis of the second messenger cGMP in the light transduction pathway of guanylate cyclase [52]. Iron mediates the signal transduction ofG-protein-coupled receptor 91 (GPR91) in retinal ganglion and RPE cells, which stimulates the secretion of VEGF [53]. The lipid-bearing disk of the outer photoreceptor segment experiences continuous synthesis and shedding, and the disk synthesis requires the participation of Ft. Therefore, iron regulation has become an important part of retinal physiology. However, the oxidation of excessive ferrous and hydrogen peroxide in the retina can initiate ROS production through the Fenton reaction (Fe^2+^ + H_2_O_2_→Fe^3+^ + HO^•^ + HO^−^) [54]. Iron homeostasis consists of the process of iron uptake, utilization, and storage. The intracellular iron level is regulated at three levels: controlling iron uptake through TfR expression, modulating the labile iron pool through Ft expression, and regulating iron export through ferroportin (Fpn) expression [55]. In systemic circulation, iron mainly exists in the forms of heme iron and non-heme iron [56]. Fe^2+^ is oxidized to Fe^3+^ by the iron oxidase Hepc or Cp, which binds to the iron transporter transferrin (Tf) [57]. In the retina, Tf is mainly expressed in RPE cells and photoreceptors [58]. In this context, trivalent iron can readily enter the RPE cells and photoreceptors with the assistance of Tf. Fe^3+^ is separated from Tf-TfR, and eventually stored in Ft or transferred to the cytoplasm under an intracellular acidic environment. In acidic inclusions, Fe^3+^ is reduced to Fe^2+^ by the six-transmembrane epithelial antigen of prostate 3 (STEAP3), and then is transported to cytoplasm by divalent metal transporter 1 (DMT1) [59]. Upon cellular uptake, Fe^2+^ may be stored in the cytoplasmic Ft or the mitochondrial ferritin (FtMt). In the cytoplasm, Fe^2+^ is released from Ft during Ft degradation and is selectively recognized by cytosolic Fe^2+^ chaperones PCBPs1/2. In the mitochondria, Fe^2+^ is recognized by the mitochondrial Fe^2+^ chaperone, frataxin. Subsequently, the recognized Fe^2+^ is made available for utilization by multiple Fe^2+^-dependent proteins, enabling their proper functioning in cellular processes. The remaining Fe^2+^ enters the labile iron pool as an active redox iron source [57]. In classic theories, the intracellular labile iron pool is used for iron storage, export, and transportation [60]. Unused iron is transported out of the cell through the action of the iron oxide enzyme [61]. In a pharmacologically induced AMD model, the Ft content increases significantly, and exclusively localizes in the RPE cell and photoreceptors [62]. Clinical research also shows that the Tf expression in the retina of AMD patients is significantly higher compared with that of age-matched healthy controls [63]. Therefore, iron homeostasis is essential for maintaining the normal retinal structure and function (Figure 2).

### 3.2. Activity of Transferrin Receptor

Controlling iron uptake by reducing the expression of TfR is a critical way to maintain the iron homeostasis [33]. Fe^3+^-Tf can enter the cell through the TfR in the cell membrane. The TfR is a membranous receptor composed of two subunits (approximately 90 KDa) that are linked to each other through disulfide bonds, resulting in a dimeric configuration. It acts as a regulator for the cellular intake of iron and performs a vital function in preserving iron equilibrium within cells [64]. The TfR family has two members, TfR1 and TfR2 [65]. In virtually all vertebrates, cells absorb and transport iron through the Tf- TfR1 system. TfR1 is a type II transmembrane glycoprotein that actively participates in the cellular uptake of iron. Conversely, TfR2, which is a homolog of TfR1, also has the ability to bind to and internalize the Tf-Fe^3+^ complex. However, TfR2 displays a lower affinity for Tf-Fe^3+^ and has a more restricted distribution compared with TfR1. TfR plays a major role in cellular iron uptake through binding and internalizing a carrier protein, Tf. Since TfR1 acts as a receptor for incorporating iron into cells, its expression is affected by the cellular iron status. In a cellular iron-deficient state, TfR1 expression increases, whereas in the presence of excess iron, TfR1 expression decreases. On the other hand, TfR2 has no iron reaction element, and its expression level is not affected by intracellular iron levels [66]. The expression of TfR1 is regulated at both transcriptional and post-transcriptional levels. Hypoxia response elements exist in the promoter of the tfr1 gene [67]. The iron regulatory protein (IRP)–iron-responsive element (IRE) system plays an important role in the post-transcriptional modulation of TfR1 expression. Under iron-deficient conditions, IRPs bind to IREs and stabilize the TfR mRNA, thereby enhancing the expression of the TfR1 protein. When excessive iron accumulates in cells, IRPs lose interaction with IREs, resulting in the destabilization and degradation of TFR mRNA. In addition, the human hemochromatosis protein (HFE) is a major histocompatibility complex class I molecule expressed in the cell membrane that competes with Fe^3+^-Tf for the binding site of TfR1. It can reduce the binding affinity of Fe^3+^-Tf [68,69]. TfR1 expression is also modulated by intracellular iron concentration. Pseudolaric acid B can enhance the expression of TfR through promoting the input of iron [70]. In light-induced 661w cells, intracellular total iron increases in a time-dependent manner, and the expression level of TfR1 mRNA increases significantly [71]. Taken together, the expression level of TfR1 increases during iron deficiency, while it decreases with excessive iron. Hence, meticulous regulation of the expression level of TfR can effectively safeguard against the detrimental impacts arising from iron deficiency or excessive iron accumulation, thereby upholding the equilibrium of iron metabolism in the cell.

### 3.3. Regulation of Ferritin

Ft is the major iron storage protein in cells that plays an important role in maintaining iron balance [72]. The iron-free form of this protein is termed apoferritin, while the iron-containing form is called holoferritin, or simply Ft. The shell of apoferritin consists of 24 subunits, and has two types of monomers [64]. The L monomer contains 174 amino acids with a molecular weight of 18,500 d, while the H monomer has 182 amino acids with a molecular weight of 21,000 d [73]. Each apoferritin can sequester up to approximately 4500 iron atoms [74]. The iron storage protein Ft includes ferritin light chain (FTL1) and ferritin heavy chain (FTH1). FTH1 has iron oxidase activity, which can convert excessive Fe^2+^ into Fe^3+^, promote the accumulation of iron ions in FTL1, and promote the antioxidant function of cells. Nuclear receptor coactivator 4 (NOCA4) mediates the Ft degradation through phagocytosis activities [75]. Ft synthesis can alter dynamically in response to changes in the intracellular iron level. For instance, Ft synthesis increases in parallel with iron accumulation. The synthesis of Ft is modulated by the IRE-mediated binding of RNA-binding proteins with stem–loop structures, specifically in the 5′ region of Ft H and L mRNA, in response to the iron level [33]. IRP is the key regulatory protein that plays a critical role in maintaining iron homeostasis. Generally, IRP exists in two forms, IRP1 and IRP2, both of which can bind to the stem–loop structure of IRE and inhibit the translation of mRNA in cells [76]. In the case of iron deficiency, the open configuration of IRP1 can combine with the stem ring of IRE to hinder the effective translation of Ft and Fpn mRNA. This adjustment is beneficial for maintaining the stability of TfR mRNA and enhancing the availability of iron within cells [77]. On the other hand, IRP1 exists in the form of cycloaconitase when the cell is exposed to excessive iron. Cycloaconitase can combine with the 4Fe-4S cluster, and the citrate is transformed into isocitrate [77,78]. IRP2 has an additional cysteine-rich 73 amino acid insertion in its N-terminus. IRP2 is ubiquitinated and degraded by the proteasome in iron-replete cells [79]. The binding of iron to IRP leads to conformational changes, which promote the transcriptional translation of Ft and Fpn. The binding activity also disrupts the stability of TfR and divalent metal ion transporter mRNA, reduces the rate of iron absorption, and increases the capacity of iron storage. Iron release from Ft is regulated by a process known as ferritinophagy. Ferritinophagy is mediated by nuclear receptor coactive 4 (NCOA4), which directly binds FTL1 and transfers the complex to the autolysosome for degradation [80].

### 3.4. Regulation of Ferroportin

Fpn is composed of 12 transmembrane helices, and it is the sole non-heme iron transporter found in mammals [81]. Iron is transported from iron storage cells to the blood continuously, thereby maintaining the dynamic balance of iron metabolism [82]. If the intracellular iron level is saturated, excess iron is transported outside the cell through Fpn. The Fe^2+^ is oxidized to Fe^3+^ through Cp and Hepc, which subsequently bind to TF (Fe^3+^-Tf) to enter the iron uptake cycle again [81]. The peptide hormone Hepc is involved in the regulation of iron homeostasis. In the retina, Hepc is expressed extensively in the photoreceptors, Müller cells, and RPE cells [83]. Hepc expression is affected by systemic stimuli such as iron storage, inflammation, hypoxia, and oxidative stress. These stimuli act through liver cell surface proteins (e.g., HFE, TfR2, IL-6R, etc.) to control Fpn levels [84]. Hepc regulates the absorption and release of iron in the body through inhibiting the open–outward conformation of Fpn, an iron efflux protein. Moreover, Hepc can induce the endocytosis and degradation of Fpn in cells. When excess iron accumulates in the body, an increased level of Hepc suppresses the functionality of Fpn, resulting in reduced iron release and absorption. On the other hand, when iron is insufficient in the body, decreased levels of Hepc allow for the increased activity of Fpn. Fpn promotes iron release and absorption to meet the iron demands of tissue [85].

## 4. Lipid Peroxidation and Ferroptosis in AMD

**Figure 3:**
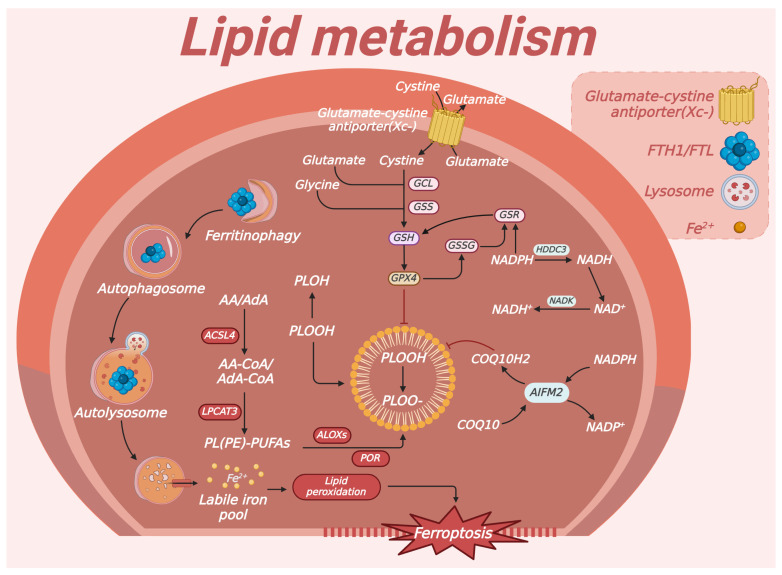
Basic signaling pathways in lipid metabolism. PUFAs are prone to generating lipid peroxyl radicals and hydroperoxides through LOX. ROS produced as a result of accumulated iron, via Fenton reactions, further interact with PUFAs facilitating lipid peroxidation. This cascade of reactions promotes ferroptosis. Some common lipophilic antioxidants include ferrostatin-1, liproxstatin-1, zileuton, and L-carnitine. PUFAs, polyunsaturated fatty acids.

Lipids are plentiful in retinas and undergo continuous recycling as lipid-rich photoreceptor outer segments are shed and regenerated (Figure 3). Fatty acid β-oxidation significantly contributes to the neural retina’s energy supply. Ferroptosis is initiated by lipid peroxidation resulting from the oxidative impairment of the antioxidant defense system [86]. Lipid peroxidation is a cascade reaction, driven by free radicals or non-radical species, and instigated by the interaction of activated ROS with PUFAs. PUFAs generate lipid peroxyl radicals and hydroperoxides via enzymes like LOX and cyclooxygenase (COX) [15]. Free iron serves as a catalyst in the lipid peroxidation process, wherein PUFAs act as substrates for iron-mediated lipid peroxidation within phospholipids (PL). The polyunsaturated acyl chains of PL are oxidized to form R by losing hydrogen to hydroxyl. R readily reacts with molecular oxygen to form peroxyradicals (R-OO) [87]. R-OO extracts hydrogen from PL molecules to form lipid hydrogen peroxide (R-OOH). R-OO can also be added to the diallyl site of another PL to form R-OO-R dimers. In the Fenton chemical process, R-OOH can undergo reductive cracking to produce alkoxy (RO) radicals that promote the production of lipid peroxides (malondialdehyde, isoprostanes, and 4-hydroxy-2-nonenal, etc.) [88]. Lipidomic studies suggest that phosphatidylethanolamines (PEs) containing arachidonic acid or its elongation product, adrenic acid, are key PLs that undergo oxidation and drive cells towards ferroptosis [86]. ROS interact with PUFA to generate lipid peroxides, a significant marker of ferroptosis. Iron amplifies lipid peroxide production via iron-dependent oxidases, such as LOX [89]. Phosphorylase kinase G2 (PHKG2) regulates the availability of iron to LOX, then initiates ferroptosis through inducing the peroxidation of PUFAs at the diallyl position. PUFA can be integrated into the membrane through the enzymes acyl-coenzyme A (CoA)–synthetase long-chain family member 4 (ACSL4), a key enzyme that can promote lipid peroxide. The peroxidation is ultimately catalyzed by lysophosphatidylcholine acyltransferase 3 (LPCAT3) after the acetylation of ACSL4 [90]. ACSL4 promotes neuronal death via enhancing lipid peroxidation, a marker of ferroptosis. Moreover, knockdown of ACSL4 inhibits pro-inflammatory cytokine production in microglia. These data identify ACSL4 as a novel regulator of neuronal death and neuroinflammation. In molecular dynamics, the presence of lipid peroxides leads to an increase in biofilm curvature, coupled with the hydrophilic nature of the acyl tail of lipid peroxides. This combination results in membrane instability, thereby influencing cellular survival [91].

The neural retina uses β-hydroxybutyrate, a ketone body produced by RPE cells from fatty acids, as a metabolic substrate, and could shield the photoreceptor from the high-stress environment of the subretinal space. Disk membranes of photoreceptor outer segments have the highest concentrations of docosahexaenoic acid (DHA), which favors protein movements and protein–protein interactions as a result of greater viscosity, and may increase the transmission efficiency of chemical signals and photic stimuli. Omega-3 long-chain polyunsaturated fatty acids (n-3 LC-PUFAs) enhance the accumulation of lutein and zeaxanthin, which not only filter blue light, but also exhibit antioxidant and anti-inflammatory properties. Indeed, the levels of omega-3 fatty acids, core components of photoreceptors, decrease significantly in AMD patients. To evaluate the association of lipid intake with baseline severity of AMD in the Age-Related Eye Disease Study (AREDS), more dietary n-3 LC-PUFAs, as components of fish-based foods, may reduce the risk of progression from bilateral drusen to central geographic atrophy in AMD patients [92]. Retinal cells rich in PUFA are highly sensitive to lipid peroxide [93]. Photoreceptor death is closely associated with disruption of all-trans retinal (atRAL) clearance in the neural retina. In vitro experiments demonstrate that atRAL disrupts iron homeostasis and causes an elevation of Fe^2+^ level in 661W cells. Furthermore, atRAL induces lipid peroxidation in 661W cells in a time-dependent manner [94]. Hence, it is crucial for preserving vision by maintaining the homeostasis of lipid metabolism and inhibiting the onset of ferroptosis.

## 5. Mitochondria Dysfunction of Ferroptosis in AMD

**Figure 4:**
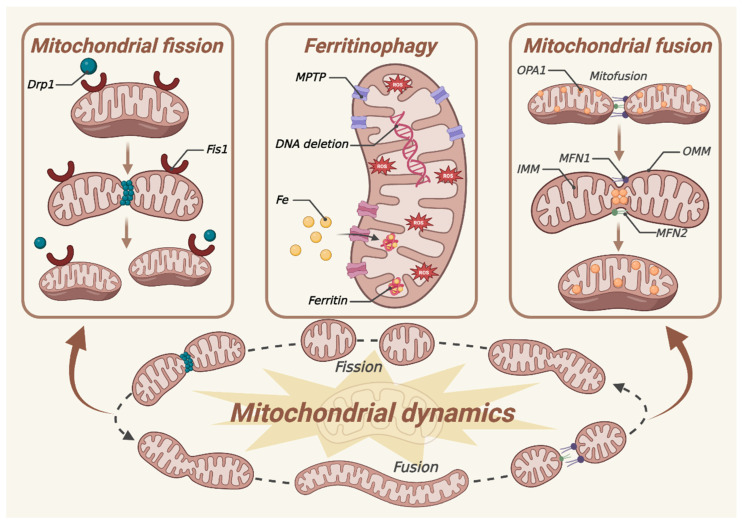
Ferroptosis causes mitochondria dysfunction during age-related macular degeneration. Mitochondrial quality control mechanisms, including fusion, fission, and mitophagy, serve to safeguard mitochondria from stress-induced harm. Multiple DRP1 molecules are recruited to a single mitochondrion, binding to these receptors and closely encircling the mitochondrion to form a ring-like structure. Subsequently, through their GTPase activity, DRP1 molecules hydrolyze GTP, inducing permeabilization of both MIM and MOM, thereby facilitating mitochondrial fission. In this fusion process, the fusion of MOM is primarily mediated by proteins such as MFN1 and MFN2. OPA1 promotes inner membrane fusion via MFN1. Ferritinophagy is associated with Ft degradation. NCOA4 promotes Ft transport and iron release to regulate iron homeostasis. Drp1, GTPase dynamin-related protein 1; IMM, inner mitochondrial membranes; OMM, outer mitochondrial membranes; MFN1, mitochondrial fusion protein 1; MFN2, mitochondrial fusion protein 2; OPA1, optic atrophy 1 protein; Ft, ferritin.

Mitochondria are semi-autonomous organelles which can self-regulate through mitochondrial quality control mechanisms, thereby supplying energy for cellular homeostasis (Figure 4). Mitochondrial quality control mechanisms, including fusion, fission, and mitophagy, serve to safeguard mitochondria from stress-induced damage [20]. These processes selectively remove damaged proteins or dysfunctional mitochondria, preserving mitochondrial morphology, structure, and function, thereby fostering cell survival. However, these cellular quality control pathways diminish with age [95]. Pathological conditions, such as mtDNA mutations, mitochondrial fusion/fission imbalance, and dysregulated mitophagy, can lead to abnormal mitochondrial morphology, oxidative phosphorylation dysfunction, reduced ATP synthesis, cytochrome C release, and the activation of mitochondria-dependent cell signaling, which in turn cause mitochondrial dysfunction, ultimately resulting in ferroptosis.

### 5.1. Mitochondrial Fission and Fusion in AMD

Mitochondrial fission contributes to quality control because it promotes the damaged mitochondria to split into one mitochondrion with normal function and one with abnormal function. Then, the abnormally functioning mitochondria are eliminated by mitophagy to maintain mitochondrial homeostasis. During this process, the GTPase dynamin-related protein 1 (DRP1) serves as a pivotal mediator, interacting with various protein receptors such as mitochondrial fission factor (Mff), mitochondrial fission protein 1 (Fis1), mitochondrial dynamics protein 49 (MiD49), and mitochondrial dynamics protein 51 (MiD51) [96]. Multiple DRP1 molecules are recruited to a single mitochondrion, binding to these receptors and closely encircling the mitochondrion to form a ring-like structure. Subsequently, through their GTPase activity, DRP1 molecules hydrolyze GTP, inducing permeabilization of both the mitochondrial inner and outer membranes (MIM and MOM), thereby facilitating mitochondrial fission. Following fission, DRP1 is redistributed to the cytosol. Mitochondrial fusion is the process of fusing two adjacent MOM and MIM to form new mitochondria, ensuring the efficient exchange of substances (mitochondrial DNA, proteins, metabolites, etc.) within mitochondria [97]. During this process, the fusion of the MOM is primarily mediated by proteins such as mitochondrial fusion protein 1 (MFN1) and mitochondrial fusion protein 2 (MFN2). The optic atrophy 1 protein (OPA1) plays a key role in mediating the fusion of the MIM, and OPA1 promotes inner membrane fusion via MFN1, but not MFN2 [98]. Mitochondria fusion produces an increase in cristae density, which results in closer respiratory complexes and a higher rate of oxidative phosphorylation. One prominent hypothesis posits that mitochondrial defects in the RPE contribute to AMD pathology. It is supported by several studies involving human retinas obtained from eye bank donors. The examination of electron microscopy images reveals that individuals with AMD exhibits a significant reduction in mitochondrial number, decreases surface area, and also causes a loss of cristae and matrix density [99]. Other studies have shown that RPE from non-exudative AMD patients has fewer and smaller mitochondria, suggesting impaired biogenesis and fusion, as well as alterations in mitochondrial membranes and cristae structure [100]. C57BL/6J mice are exposed to blue light to induce the AMD model. Prolonged exposure to blue light significantly reduces the thickness of each retinal layer and results in impaired retinal mitochondria. The findings reveal a significant increase in DRP1 protein levels, and a decrease in OPA1 and Bcl-2 protein levels. These results suggest that mitochondrial dynamics are disrupted, showing characteristics of fusion-related obstruction following blue light irradiation [101]. Hence, precise modulation of alterations in mitochondrial dynamics can impact mitochondrial function and retard the onset of ferroptosis, thus presenting novel avenues for AMD therapeutics.

### 5.2. Ferritinophagy in AMD

Ferritinophagy, a newly discovered form of autophagy associated with Ft degradation, is facilitated by NCOA4. It promotes Ft transport and iron release, as needed to regulate iron homeostasis. NCOA4 finely regulates cellular iron homeostasis by anticipating the autophagic degradation of Ft. At the initial phase of ferritinophagy, the interaction between the subunits of Ft (FTH1/FTL1), facilitates a strong binding with the C-terminal domain of NCOA4. This process is mediating by an immediate interplay with conserved surface arginine residues. Subsequently, HECT and RLD domain-containing E3 ubiquitin protein ligase 2 (HERC2) establishes close contact with the NCOA4–Ft complex following the initiation of ferritinophagy, thereby enhancing functional stability [102]. Under iron-replete cellular conditions, HERC2-mediated ubiquitylation facilitates the turnover of NCOA4. HERC2 binds more strongly to NCOA4, thereby augmenting proteasomal degradation of NCOA4. However, FTH1 is another mediator that facilitates the dissociation of the NCOA4–FTH1 complex under iron-replete cellular conditions, eventually contributing to the suppression of ferritinophagy and Ft iron reservation [103]. Under iron-deficient cellular conditions, NCOA4 is stabilized to promote ferritinophagy. NCOA4 facilitates the formation of autophagosomes, and directs it to the lysosome for degradation, which in turn increases cellular iron levels [104]. In a pathologically high intraocular pressure (ph-IOP) injury model induced by saline perfusion into the mouse anterior chamber, there is an abnormal accumulation of Fe^2+^ in the retina, along with elevated serum iron levels post-injury. Knockdown of ncoa4 in mice results in reduced FTH1 degradation and retinal ferrous iron level [105]. NCOA4-mediated ferritinophagy contributes to the maintenance of mitochondrial functions through supplying iron to mitochondria. A notable increase in intracellular iron level is detected in model of senescent ARPE-19 cells induced by tert-butyl hydroperoxide (tBH), accompanied by a rise in NCOA4 expression over time and a concurrent decrease in Ft level. Remarkably, NCOA4 expression positively correlates with the essential autophagy-related protein LC3B. Additionally, it is noteworthy that iron overload contributes to mitochondrial dysfunction [106]. Mitophagy is an important process related to ROS metabolism. ROS are produced through the mitochondrial electron transport chain (ETC) in normal metabolism, which can maintain the balance of the body through the rapid elimination of antioxidant stress metabolism, such as SOD and GSH [107]. ROS, such as superoxide anion, hydroxyl radical, and hydrogen peroxide, play a dominant role in mediating oxidative damage [48]. There is evidence that transient mitochondrial permeability transition pore (mPTP) openings are crucial for maintaining healthy mitochondrial homeostasis. Adaptations and adverse reactions to redox stress may involve the activation of mitochondrial channels, which cause internal and intermittent redox environmental changes, leading to ROS release. At higher ROS levels, longer mPTP openings may release ROS bursts and cause mitochondrial destruction [108,109].

## 6. The Regulatory Role of Ferroptosis in AMD

**Figure 5:**
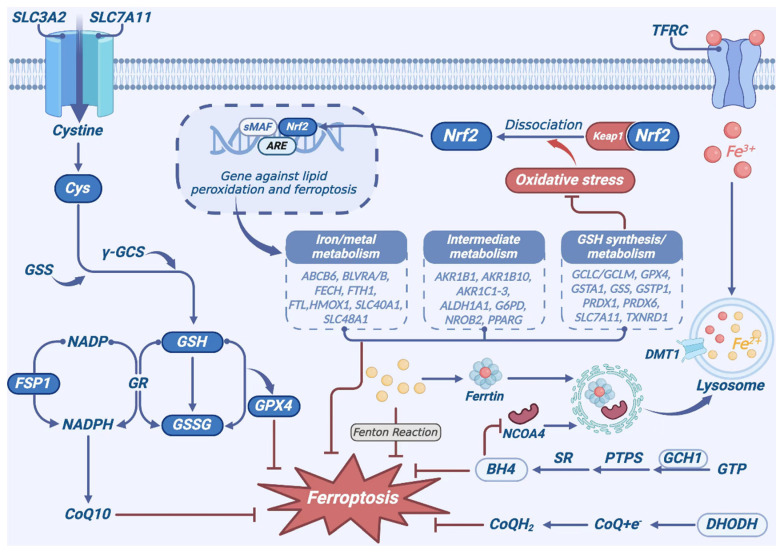
Ferroptosis-related signaling pathways that significantly hinder the progression of AMD. These pathways include the GSH-GPX4 regulating axis, GCH1-BH4 regulating axis, FSP1-regulating pathway, DOHDH–CoQH2 pathway, and Nrf2-regulating pathway. These pathways collectively play crucial roles in regulating iron overload and lipid peroxidation, thereby mitigating the pathological processes associated with AMD. SLC7A11, Subunits cystine/glutamate antiporter solute carrier family 7 member 11; SLC3A2, solute carrier family 3 member A2; Cys, cysteine; GSS, glutathione synthetase; γ-GCS, γ-glutamylcysteine synthase; FSP1, ferroptosis suppressor protein 1; GR, glutathione reductase; GSH, glutathione; GSSG, glutathione disulfide; GPX4, glutathione peroxidase 4; Keap1, Kelch-like ECH-associated protein 1; Nrf2, nuclear factor erythroid 2-related factor 2; TFRC, transferrin receptor; DMT1, divalent metal transporter 1; NCOA4, nuclear receptor coactive 4; SR, sepiapterin reductase; PTPS, protein tyrosine phosphatases; DHODH, dihydroorotate dehydrogenase; BH4, tetrahydrobiopterin; ABCB6, ATP-binding cassette transporter B6; BLVRA/B, biliverdin reductase A/B; FECH, ferrochelatase; HMOX1, heme oxygenase-1; FECH, ferrochelatase; SLC48A1, solute carrier family 48 member A1; AKR1B1, aldo–keto reductase family 1 member B1; AKR1B10, aldo–keto reductase family 1 member B10; AKR1C1-3, aldo–keto reductase 1C 1-3; ALDH1A1, aldehyde dehydrogenase 1A1; G6PD, glucose-6-phosphate dehydrogenase; NROB2, nuclear receptor small heterodimer partner; PPARG, peroxisome proliferator-activated receptor-gamma; GCLC/GCLM, catalytic and regulatory subunits of glutamate cysteine ligase; GSTA1, glutathione S-transferase alpha 1; GSS, glutathione synthetase; GSTP1, glutathione S-transferase pi 1; PRDX1, peroxiredoxin 1; PRDX6, peroxiredoxin 6; TXNRD1, thioredoxin reductase.

During the process of ferroptosis, pathogenesis alterations such as lipid metabolism, iron accumulation, and oxidative stress profoundly affect normal visual function. Extensive studies have established that targeted ferroptosis-related signaling pathways markedly impede the progression of AMD through multi-facial mechanisms, such as the GSH-GPX4 pathway, the FSP1–CoQ10–NADH pathway, dihydroorotate dehydrogenase (DHODH)–dihydroubiquione (CoQH2) pathway and GTP cyclohydrolase1–tetrahydrobiopterin (GCH1-BH4) axis, etc. [110]. Accumulating evidence shows that inhibiting ferroptosis enhances retinal cell survival, and may develop into an effective therapeutic strategy for AMD (Figure 5).

### 6.1. GSH-GPX4 Regulating Axis

GPX4 is a cysteinase-containing and GSH-dependent enzyme that can reduce phospholipid hydroperoxide and cholesterol hydroperoxide to their counterparts, thus interrupting the chain reaction of lipid peroxides. Furthermore, it can protect the liposomes and biofilms of phosphatidylcholine from oxidative degradation [111]. The function of GPX4 decreases with the increase in free iron utilization rate and PUFA oxidation, which is considered as a sign of ferroptosis [112]. Under the condition of reduction, cystine is directly absorbed by the alanine, serine, and cystine selective system (System ASC). After cystine enters the cell, it is reduced to cystathionine by GSH or thioredoxin reductase (TXNRD1), which produces GSH catalyzed by γ-glutamylcysteine synthase (γ-GCS) and GSH synthetase (GSS) [20]. Two GSH molecules can be used as electron donors to reduce phospholipid peroxides (PL-OOH) to corresponding alcohols under the action of GPX4 and to form GSSG at the same time. GSSG can be reduced to GSH by GSH reductase using NADPH [26]. GSH reduces toxic phospholipid hydroperoxides (PE-AA-OOH/PE-AdA-OOH) to an electron donor of non-toxic phospholipid alcohol (PE-AA-OH, PE-AdA-OH), and GSSG can be regenerated by reducing GSSG with NADPH, which acts as an electron donor [113,114]. GSH is the substrate of the GPX4 catalytic reaction and is involved in the regulation of ferroptosis [112]. The biosynthesis of GSH involves the amino acids cysteine, glycine, and glutamic acid, with cysteine being the rate-limiting factor. Targeting these sites to inhibit the synthesis of GSH would be effective to induce ferroptosis. The heterodimeric amino acid transporter system Xc− is composed of SLC7A11 and SLC3A2 subunits. It is responsible for the cellular uptake of oxidized cysteine [26]. The cystine/glutamate anti-transporter SLC7A11 (also known as xCT) is designed to introduce cystine for GSH biosynthesis and antioxidant defense [115]. Cystine is transported into cells, glutamic acid is exchanged, and GSH production is maintained. Under the condition of oxidation, system Xc- is the only transporter that can provide cystine and cystathionine from the extracellular membrane, and exchanges glutamic acid outside the cell membrane. This is the upstream event of ferroptosis cascade reaction [75]. The expression of SLC7A11 is a key component of the GSH biosynthesis pathway. It is tightly regulated by Nrf2 and BRCA1-associated protein 1 (BAP1). Nrf2 is a transcription factor that activates the expression of antioxidant response genes, including SLC7A11, to maintain cellular redox balance. BAP1 interacts with Nrf2 to modulate SLC7A11 expression and GSH levels [116,117,118]. It has been found that erastin inhibits the uptake of cystine by system Xc- and leads to cell death [10]. The expression of SLC7A11 and GPX4 protein is significantly decreased in light-induced retinal degeneration (RD) mice [119]. In a laser-induced choroidal neovascularization (CNV) mouse model, intraperitoneal injection of the SLC7A11 inhibitor expands CNV area. In the ARPE19 cell line, SLC7A11 inhibition or SLC7A11 knockdown increases lipid peroxidation levels and decreases the cell viability of ARPE19 cells [13]. Ferroptosis has been detected in various experimental models, including human primary retinal pigment epithelial cells (HRPEpiC) and RD mice induced by NaIO_3_. Moreover, GSH-GPX4 signaling is impaired after NaIO_3_-induced toxic damage in RPE cells, which is exacerbated by the silencing of GPX4 [120]. In the dry AMD mice model induced by NaIO_3_, both lipid peroxidation and transmission electron microscopy results reveal a significant activation of ferroptosis. This is evidenced by the observation of smaller mitochondria with increased membrane density in RPE and choroidal tissue. Additionally, there is a decrease in the expression of both FTH1 and GPX4 proteins [121]. The induction of oxidative stress-induced ferroptosis in 661W cells is achieved using H_2_O_2_. The outcomes reveal an increase in oxidative stress markers such as P53 and ALOX12, coupled with a decrease in the expression levels of SLC7A11 and GPX4 [122]. Light exposure significantly decreases the viability of 661w cells in vitro and triggers pro-ferroptotic alterations, such as iron accumulation, mitochondrial shrinkage, depletion of GSH, elevated levels of malondialdehyde (MDA), and reduced protein expression of SLC7A11 and GPX4 [119]. In in vitro studies, the immortalized rat retinal precursor cell line R28 is transfected with siRNA-targeting GPX4 for specific knockdown. The results reveal a significant increase in lactate dehydrogenase activity, with a 13.9-fold and 3.2-fold elevation in peroxidized lipid levels in R28 cells following GPX4 suppression. Defective GPX4 expression intensifies cytotoxicity, underscoring its crucial role in cellular protection [123]. These results highlight the crucial involvement of GPX4 in ferroptosis during AMD.

### 6.2. GCH1–BH4-Regulating Axis

BH4 is a redox-active cofactor that possesses antioxidant properties. It is involved in the production of nitric oxide, neurotransmitters, and aromatic amino acids. BH4 is paired with dihydrobiopterin (BH2), and forms a redox cycle to diminish endogenous oxidative radicals and protect lipid membranes to inhibit ferroptosis. Metabolic control of BH4 levels is tight and controlled by three main pathways: the de novo synthesis cascade, the circulation pathway, and the salvage pathway. In the de novo pathway, the biosynthesis of BH4 from its precursor GTP requires three enzymatic steps catalyzed by GCH1, 6-pyruboyltetrahydropterin synthase (PTS), and sepiapterin reductase (SPR), among which the GCH1 is the rate-limiting enzyme [124]. Genetic or pharmacological inhibition of GCH1 leads to BH4 insufficiency, promoting cellular peroxidation accumulation and susceptibility to ferroptosis. The expression levels of GCH1 significantly determine the extent of cell resistance to ferroptosis [125]. In the circulation pathway, GCH1 converts GTP into 7, 8-dihydropterate triphosphate when SPR is deficient. Subsequently, under the catalytic reactions of aldehyde reductase and carbonyl reductase mediated by PTS, BH2 is formed, which is then converted into BH4 by dihydrofolate reductase. In the salvage pathway, quinone-BH2 is conversed into BH4 under the action of quinone-BH2 reductase [126]. The whole genome library CRISPR/Cas9 screening shows that GCH1 suppresses ferroptosis. GCH1 overexpression in mouse fibroblasts significantly inhibits ferroptosis induction by RSL-3 treatment and GPX4 inhibition [127]. Research indicates that BH4 deficiency increases sensitivity to Parkinson’s disease (PD)-related stressors, which triggers lipid peroxidation and ferroptosis in midbrain dopaminergic neurons in a mouse model of PD. Genetic and pharmacological augmentation of BH4 can independently regulate tyrosine hydroxylase levels, prevent ferroptosis, mitigate mitochondrial ROS accumulation, and sustain neuronal excitability [128]. Oxidative stress, mitochondrial dysfunction, and neuroinflammation are key contributors to ferroptosis. In PD, BH4 has been shown to play a protective role by modulating oxidative stress and mitochondrial function. These findings suggest that BH4 may similarly influence oxidative stress pathways in AMD, potentially contributing to ferroptosis in retinal cells. Therefore, although the direct link between BH4 and ferroptosis in AMD remains unexplored, the similarity between these diseases provides a rational basis for further investigation into the role of BH4 in AMD-related ferroptosis.

### 6.3. FSP1- Regulating Pathway

Ferroptosis suppressor protein 1 (FSP1) is a flavin protein redox reductase characterized by a short N-terminal hydrophobic sequence and a typical flavin adenine dinucleotide (FAD)-dependent oxidoreductase enzyme domain [129]. Its primary function is to acylate and bind to motifs in the plasma membrane, thereby effectively trapping antioxidants to counteract lipid peroxidation [130]. FSP1 features an N-terminal myristoylation, which is critical for targeting FSP1 to lipid droplets and the plasma membrane. FSP1 prevents ferroptosis by converting ubiquinone-10 (CoQ_10_) to ubiquinol-10 (CoQ_10_H_2_) on the plasma membrane. This process is facilitated by N-myristoylation, with NAD(P)H acting as the substrate. This process reduces the levels of oxidized CoQ10, effectively depleting the pool of oxidized CoQ_10_. Acting akin to a lipophilic radical-trapping antioxidant (RTA), FSP1 halts the propagation of lipid peroxides, thereby thwarting ferroptosis [131]. CoQ_10_ is composed of a benzoquinone ring and a polyisoprenoid tail containing between 6 and 10 subunits that are species-specific, and which is an effective lipophilic antioxidant, able to capture lipid peroxide free radicals [132]. CoQ_10_ occurs in all tissue in the animal organism and shows a potent antioxidant function in plasma membranes, endomembrane, and lipoproteins. Meanwhile, CoQ_10_ is the main effector of the FSP1 pathway [133]. The CoQ_10_ biosynthesis pathway is tightly regulated at both transcriptional and translational levels. Genomic CRISPR/Cas-mediated activators allow the identification of rate-limiting enzyme GCH1 for the synthesis of antioxidant BH4 [129,134]. BH4 can prevent the consumption of CoQ_10_ by reducing oxidative stress, or convert phenylalanine to tyrosine to promote the synthesis of CoQ, thus maintaining the level of CoQ_10_ [112]. Moreover, oral administration of CoQ_10_ is reported in the treatment of PD and diabetes [135]. Interestingly, FSP1 may play a significant role in mediating mitochondrial stress. When oxidative stress occurs, FSP1 binds with 4-hydroxy-2-nonenal (HNE), a lipid peroxidation end product, forming a lipid adduct that lacks oxidoreductase activity. The HNE-FSP1 adduct is then transported from the mitochondria to the nucleus, leading to DNA damage and cell death [136]. FSP1, along with electron donors NAD(P)H/H+, has been identified as a second system that inhibits iron oxidation which effectively blocks lipid peroxidation independently of the GSH-GPX4 axis [118]. ARPE-19 cells administered by NaIO_3_ shows decreased cell vitality and serious mitochondrial damage. The decline in cell viability is alleviated in cells overexpressed with FSP1, suggesting that FSP1 has a protective effect against NaIO_3_ toxicity [120].

### 6.4. DOHDH–CoQH_2_ Pathway

As a flavin-dependent mitochondrial enzyme, DHODH is directly coupled to the ETC within mitochondria. It facilitates the transfer of electrons to CoQ, resulting in the conversion of CoQ to CoQH_2_, which possesses antioxidant properties and inhibits the formation of lipid peroxides. The inhibition of DHODH expression leads to impaired functionality of the ETC complex, resulting in disrupted oxidative phosphorylation in cells [137]. This disruption culminates in the accumulation of lipid peroxides and mitochondrial iron-induced cell death. DHODH has been identified as a defense mechanism against ferroptosis in mitochondria, and is currently being investigated in studies aimed to inhibit the proliferation of tumor cells [138]. In a mouse model of subarachnoid hemorrhage (SAH), intraperitoneal injection of vitamin K_2_ subtype menaquinone-4 (MK-4), known for its protective role in mitochondrial function, significantly increases DHODH protein levels. This leads to a reduction in ROS, restoration of mitochondrial membrane potential, preservation of the neuronal mitochondrial structure, and ultimately attenuated ferroptosis [139].

### 6.5. Nrf2-Regulating Pathway

Nrf2 is a member of the basic leucine zipper transcription factor family. It acts as a key regulatory factor in cellular antioxidant responses through controlling the expression of antioxidant and electrophile response genes [140]. Nrf2 interacts with Keap1 on cytoplasmic actin through its Neh2 domain. The BTB domain of Keap1 can bind to the ubiquitin ligase Cullin3 to form an E3 ubiquitin ligase complex, leading to ubiquitination and proteasomal degradation of Nrf2. Under oxidative stress, the conformation of Keap1 changes, promoting Nrf2 to dissociate from Keap1. The dissociated Nrf2 translocates into the nucleus, where it forms heterodimers with small Maf proteins via its Neh1 domain, and binds to the promoter region of antioxidant response elements (AREs), thereby promoting the transcription of downstream antioxidant response genes [141]. Nrf2 is responsible for regulating antioxidant genes, such as GSH peroxidase, GSH reductase, superoxide dismutase, heme oxygenase-1(HMOX1), etc. [142]. Importantly, almost all genes involved in ferroptosis are regulated by Nrf2 transcription, and which can indirectly regulate lipid abundance [143]. In iron metabolism, Nrf2 regulates the FTL1/FTH1, the primary iron storage protein, as well as Fpn, which facilitates iron efflux from the cell. Nrf2 has the ability to upregulate HMOX1, which catalyzes the conversion of heme to biliverdin. The essential components involved in heme synthesis include iron chelatase (FECH) and ATP-binding cassette subfamily B member 6 (ABCB6). Furthermore, heme transporters, solute carrier family 48 member A1 (SLC48A1) and biliverdin reductase A and B (BLVRA/B), are also upregulated by Nrf2. Nrf2 targets are involved in lipid metabolism, including the small heterodimer partner (SHP), peroxisome proliferator-activated receptor γ, aldo-ketoreductases (AKR1C1–3, AKR1B1 and AKR1B10), the oxidation of aldehydes to their carboxylic acid form (aldehyde dehydrogenase 1 family member A1, ALDH1A1), and glucose metabolism/NAPDH regeneration (glucose-6-phosphate dehydrogenase, G6PD) [144]. Moreover, Nrf2 governs the activity of enzymes involved in GSH synthesis and metabolism, including the catalytic and regulatory subunits of glutamate cysteine ligase (GCLC/GCLM), GSS, and SLC7A11. Numerous oxidoreductases utilize either GSH or NADPH to reduce oxidant substrates, such as glutathione S-transferase pi 1 (GSTP1) and glutathione S-transferase alpha 1 (GSTA1). Peroxiredoxin 1 and 6 (PRDX1 and PRDX6) and thioredoxin reductase (TXNRD1) also fall under the regulatory purview of Nrf2 [143,145]. In a model of iron overload-induced RD, established via intraperitoneal injection of iron dextran in Kunming mice, excessive iron deposition significantly exacerbates the retinopathy. Puerarin activation of the Nrf2 pathway leads to increased protein expression of Nrf2, GPX4, SLC7A11, and HO-1, thereby mitigating retinal lipid peroxidation and reducing ROS production. Nrf2 activation enhances the cell’s resistance to the occurrence of ferroptosis [146]. Oxidative stress-induced damage to RPE cells is a major contributor to the pathogenesis of dry AMD. For example, the pro-oxidant agent tert-butyl hydroperoxide (t-BHP) is used to promote oxidative damage in RPE cells. Ginkgo biloba extract (GBE) pre-treatment attenuates the increase in lipid peroxidation and ferroptosis, increases the cell viability, and protects human RPE cells from oxidative injury by modulating the ERK1/2-Nrf2 axis [147]. In addition to antioxidant responses, Nrf2 is involved in other cellular processes such as autophagy, intermediate metabolism, stem cell stasis, and unfolded protein responses [148]. Nrf2 binds to the promoter region of p62, which encodes an LIR motif facilitating interaction with LC3, thereby orchestrating mitophagy in response to oxidative stress [149]. Nrf2^-/-^ mice exhibit swollen mitochondria in RPE, which is accompanied by increased autophagy-related vacuoles, and damaged mitochondria are often found adjacent to autophagy vacuoles. In addition, Nrf2^-/-^ mice exhibit typical features of RD, such as accumulated drusen and lipofuscin, as well as increased inflammatory proteins [150]. Consequently, Nrf2 plays an important role in regulating the response of ferroptosis.

## 7. Potential Therapeutic Effects Against AMD

Recently, ferroptosis has emerged as a significant focus in the treatment of various diseases. AMD patients suffer from visual loss, but their treatment is limited and the pathogenesis is still poorly known. It has been proposed that modulating the ferroptosis holds promise for AMD therapy. Targeting various pathways involved in ferroptosis, such as inhibiting the Xc- and GPX4 systems, lipid peroxidation, iron accumulation, and mitochondrial dysfunction, bears therapeutic significance. Given the pivotal roles of iron and lipid peroxides in ferroptosis, the treatment strategy for AMD involves classifying ferroptosis inhibitors into two categories: iron-chelating agent and lipophilic antioxidants (Table 1).

### 7.1. Iron-Chelating Agent

Iron chelators exhibit strong affinity for iron ions, forming complexes through functional groups within the ligand. This process effectively sequesters free iron, preventing the generation of free radicals. Consequently, iron chelators may be effective in safeguarding the retina from oxidative damage induced by excess iron. Deferoxamine (DFO), deferiprone (DFP), and deferasirox (DFX) are widely used iron chelators. DFO is the inaugural iron chelator sanctioned by the Food and Drug Administration (FDA), employed for managing acute iron intoxication and chronic iron overload in individuals afflicted with transfusion-dependent anemias. As a potent hydrophilic iron chelator, DFO boasts multiple coordination groups, facilitating its efficient binding to iron ions and formation of complexes [166]. DFO effectively mitigates lipid peroxidation, prevents GSH depletion, and decreases Fe^2+^ accumulation in the tBH-administered ARPE-19 cells. These actions significantly preserve the viability of ARPE-19 cells, highlighting the protective efficacy of DFO against oxidative stress-induced damage [151]. ARPE-19 cells’ exposure to blue light results in a surge of ROS, accompanied by a notable increase in intracellular Fe^2+^ levels. Treatment with DFO markedly ameliorates blue light-induced lipid peroxidation and enhances cell viability in ARPE-19 cells by modulating the GSH-GPX4 and FSP1-CoQ10-NADH defense systems [167]. Zinc deferriamine (Zn/DFO) constitutes the zinc complex of DFO, characterized by a spherical structure facilitating efficient cell membrane penetration. Upon cellular entry, the DFO component acts as a selective, high-affinity iron chelator, preferentially binding to unstable and oxidized Fe^2+^. Concurrently, the release of REDOX inert zinc ions from the complex further competes for ferrous binding sites. This dual mechanism effectively regulates the levels of Fe^2+^, thereby attenuating ROS generation via the Fenton reaction. Research indicates that intraperitoneal administration of Zn/DFO in rd10 mice effectively reduces retinal iron content, lipid peroxidation, and oxidative DNA damages. Following treatment, the structural integrity of photoreceptors is preserved, accompanied by a marked improvement in electroretinographic responses in the mice [155]. DFP stands as an FDA-approved, orally active iron chelator that is currently used clinically for the treatment of iron-overload, especially in thalassemia major. It exhibits affinity towards both Fe^3+^ and Fe^2+^, forming a 3:1 complex with iron, subsequently facilitating their excretion through urine [168]. Hepc is widely expressed in many tissues, including the photoreceptors, Müller cells, and RPE of the retina. Hepc binds to Fpn, triggering its internalization and lysosomal-dependent degradation. Mice deficient in the ferroxidases Cp and Hepc have retinal iron accumulation and degeneration with features of AMD. Long-term treatment with the oral iron chelator DFP in Hepc KO mice reduced both retinal and RPE iron levels, alleviating oxidative stress and preserving photoreceptor and RPE cell integrity. Moreover, retinas show a significant increase in rhodopsin mRNA levels, indicative of DFP-enhanced protection against RD resulting from chronic systemic iron overload in Hepc KO mice [152]. Cp/hephaestin double-knockout (DKO) mice with age-dependent retinal iron accumulation and some features of AMD are used to test the therapeutic effects of oral iron chelator DFP. Over 8 months, DFP mitigates retinal iron levels to 72% of untreated mice, diminishes retinal oxidative stress to 70% of the untreated level, and markedly ameliorates RD [169]. In a light-induced mouse model of RD, oral administration of DFP leads to decreased expression levels of HO-1 mRNA and the complement gene C3 in the retina. DFP administration subsequently mitigates light-induced nitrosative stress, diminishes microglial invasion, bolsters photoreceptor survival, alleviates retinal oxidative stress, and hinders the progression of RD [170]. In an NaIO_3_-induced AMD mouse model and an RD6 mutation-induced inherited RD mouse model, oral administration of DFP markedly decreases expression levels of the oxidative stress-related gene HMOX1 and the complement gene C3. Concurrently, it upregulates mRNA expression of the visual cycle genes such as rhodopsin and RPE-specific 65kDa (Rpe65). Furthermore, DFP exhibits photoreceptor protection in rd6 mice, leading to an increase in ONL thickness [153]. DFX is an iron-chelating agent that binds iron in a 2:1 ratio to forms a stable complex. In the retinal injury model of rats injected with N-methyl-D-aspartate (NMDA) in the vitreous cavity, NMDA triggers an overload of Fe^2+^ in retinal ganglion cells, and the retinal ganglion cells are lost 7 days after NMDA administration. Subsequent intraperitoneal injection of DFX reduces iron content and oxidative stress in the retina, thereby protecting retinal neurons from excitatory neurotoxicity [154].

Salicylaldehyde isonicotinoyl hydrazine (SIH) is a tridentate iron chelator that easily penetrates cell membranes and predominantly firmly binds Fe^3+^ in a 2:1 ratio. It is shown that SIH effectively mitigates unstable iron levels and reduces ROS levels in the H_2_O_2_-administrated ARPE-19 cells through iron chelation. This mechanism confers protection against oxidative stress-induced damage, ultimately enhancing cell survival [156]. In addition, SIH plays a protective role in hepatocyte HepG2 by regulating the balance between antioxidant and pro-oxidative properties by activating Nrf2 signaling [171]. The multi-target iron-chelating compound 5-[4-(2-hydroxyethyl) piperazine-1-ylmethyl]-quinoline-8-ol (VK-28) is a novel brain permeable neuroprotective iron chelator. Its two chimeric derivatives, 5-[N-methyl-N-propargylaminomethyl]-8-hydroxyquinoline (M30) and 5-[2-(methyl-prop-2-ynyl-amino)-ethyl]-quinolin-8-ol dihydrochloride (VAR10303), have been developed as a valuable therapeutic approach for AD, PD, and ALS. Recent findings reveal that VK28, M30, and VAR10303 exhibit significant downregulation of Tf, inhibit microglia activation, and reduce the production of pro-inflammatory cytokines TNF-α and IL-1β in rd10 mouse retinas. These outcomes suggest that the three iron-chelating compounds effectively regulate impaired retinal iron homeostasis, countering photoreceptor degeneration induced by iron overload in RP. VK28, M30, and VAR10303 have demonstrated a range of neuroprotective and neurorestorative effects, leading to histological and functional preservation of photoreceptors and improved visual behaviors in rd10 mice [157]. Tang et al. [158] have devised a novel method for fabricating uniform calcium ion-doped Prussian blue nanoparticles, denoted as KCa[FeIII(CN)6] (CaPB), via a convenient polymer-instructed nucleation process. These nanoparticles are modified with biocompatible polyvinyl pyrrolidone on the surface, exhibiting the ability to exchange calcium ions within the lattice with ferrous ions. Furthermore, the synthesized CaPB nanoparticles display significant SOD activity and catalase-like catalytic activity, enabling the elimination of superoxide anion radicals and H_2_O_2_. In a mouse model of RD induced by NaIO_3_, intravitreal injection of CaPB effectively halts the degeneration of RPE cells and photoreceptors. This study indicates that CaPB holds promise as a nanomedical treatment strategy tailored to target the underlying ferroptosis in AMD pathogenesis. Peroxisome proliferator-activated receptors (PPARs) serve as crucial nuclear receptors, governing the transcription of genes pivotal in lipid metabolism regulation. Fenofibrate, a PPARα agonist, also acts as an iron chelator. ARPE-19 cells treated with fenofibrate have normalized expressions of TfR1 and Ft, altered by ferric ammonium citrate (FAC) induction. Fenofibrate binds to Fe^2+^, thereby reducing intracellular iron levels and inhibiting ROS generation through downregulating the Wnt/β-catenin-signaling pathway [159]. These findings suggest that chelated iron could play a crucial role in promoting cell survival, regulating iron levels, and modulating energy metabolism in the nervous system. This underscores the potential of iron chelation as an ideal therapeutic approach for AMD.

### 7.2. Lipophilic Antioxidant

The outer segment of the photoreceptor contains high concentrations of unsaturated fatty acids, rendering the retina highly susceptible to lipid peroxidation with aging. This process is a crucial initiator of ferroptosis, and cellular lipophilic antioxidants inhibit ferroptosis by intercepting lipid peroxidation and capturing free radicals. Thus, alongside iron-chelating agents, lipophilic antioxidants such as ferrostatin-1 (Fer-1), liproxstatin-1 (Lip-1), and zileuton can also disrupt the lipid peroxidation cascade, thereby rescuing cells from ferroptosis demise. Fer-1 serves a protective function by scavenging lipid hydrogen peroxide, and acts as a potent inhibitor of ferroptosis. NaIO_3_ directly oxidizes unsaturated fatty acids within ARPE-19 cells, leading to lipid oxidative damage. However, the lipid antioxidant Fer-1 notably enhances cell survival [160]. NaIO_3_-induced primary HRPEpiC cells’ death in vitro, manifesting significant morphological alterations including ruptured cell membranes, cell debris, reduced cell density, diminished mitochondria size accompanied by heightened membrane density, and exhibiting typical ferroptosis-related morphological characteristics. Nevertheless, HRPEpiC cells pre-treated with Fer-1 exhibit reduced expression of the lipid peroxidation marker ACSL4 and enhanced cell viability. These protective effects are mediated by the GSH-GPX4 pathway [120]. Notably, 661W cells exposed to high glucose (HG) display a time-dependent reduction in cell viability. HG stimulation also leads to a significant increase in total iron levels and the lipid peroxidation product MDA, while concurrently causing a notable decrease in GSH level over time. However, treatment with Fer-1 markedly reduced ROS and MDA content, upregulated protein expressions of GPX4 and xCT, and downregulated expressions of ACSL4, FTH1, and NCOA4. Fer-1 effectively reverses HG-induced ferroptosis and increases the viability of photoreceptors [161]. HG is demonstrated to induce ARPE-19 RPE cell death, with elevated levels of ROS and GSSG, and intensified lipid peroxidation density in the mitochondrial membrane. The addition of Fer-1 to RPE cells augments cellular antioxidant capacity, leading to a reduction in cell death rates and alleviation of ferroptosis [163]. Lip-1 is a spiropyran compound that acts as a lipid peroxidation scavenger. Application of an SLC7A11 inhibitor or SLC7A11 knockdown results in elevated lipid peroxidation and reduces the viability of ARPE-19 cells, whereas Lip-1 rescues the ARPE-19 cells from oxidative stress [13]. Excessive ROS result in increased metabolites of LOX, which react with ROS to induce lipid peroxidation and ferroptosis. Zileuton is a compound known for its neuroprotective properties. It functions through inhibiting 5-lipoxygenase (5-LOX), thus averting glutamate-induced HT22 mouse neuronal cell death [172]. In the ARPE-19 oxidative stress model triggered by NaIO_3_, inhibiting 5-LOX with the specific inhibitor Zileuton or through siRNA knockdown of ALXO5 alleviates lipid peroxidation, mitochondrial damage, DNA impairment, and cell death in NaIO_3_-administered ARPE-19 cells. Moreover, in the AMD mouse model, Zileuton reduces NaIO_3_-induced lipid peroxidation in RPE cells, photoreceptor loss, and inflammatory responses by modulating 5-LOX activity [162]. Astragaloside IV (AS-IV) (C41H68O14) is a highly pure natural compound extracted from Astragalus membranaceous, possessing various biological activities including enhancing the immune system and anti-apoptotic, anti-stress, and antioxidant properties. in vitro study investigating HG-induced damage to ARPE-19 cells, AS-IV demonstrates its ability to mitigate the effects of HG. It achieves this by upregulating the expression of mir-138-5p in ARPE-19 cells, which in turn promotes the expression of Sirt1 and Nrf2 in the nucleus. Consequently, this leads to an increase in cellular antioxidant capacity and alleviated ferroptosis [163]. L-carnitine (LC) is also a potent antioxidant (free radical scavenger) that protects tissues from oxidative damage [173]. In in vitro experiments on H_2_O_2_-induced RPE cells, LC treatment restores the antioxidant capacity of RPE, as demonstrated by an increase in GSH and SOD activities. LC can protect RPE cells from oxidative damage induced by H_2_O_2_ [162]. Ommochromes are aromatic compounds that react with free radicals to prevent the oxidation of lipid molecules [165]. Erastin is a prototypical inducer of ferroptosis. In erastin-induced ARPE-19 cells, ommochromes mitigated the levels of inflammatory cytokines IL-6 and IL-8, as well as oxidative stress. Additionally, ommochromes exhibit protective effects against erastin-induced ferroptosis [174]. Hence, inhibiting ferroptosis can be achieved by lowering iron levels or enhancing antioxidant defenses. These findings offer valuable insights for developing clinical interventions.

The limitations in current therapeutic strategies for AMD should be carefully addressed. Although emerging research has highlighted the potential role of ferroptosis, inhibitors targeting this form of cell death may not fully prevent retinal cell demise due to the complexity of AMD pathogenesis, as it involves various forms of cell death, such as apoptosis, pyroptosis, and necroptosis. This suggests that a more comprehensive approach is necessary, as ferroptosis inhibitors alone may not be sufficient to halt the progression of AMD. Furthermore, oxidative stress, which is intricately linked to multiple cell death pathways, complicates the efforts to assess therapeutic efficacy based solely on ferroptosis inhibition. Additionally, lipid peroxides, which play a crucial role in modulating immune responses, also contribute to AMD pathogenesis. This underscores the need for strategies that not only target ferroptosis, but also address oxidative stress, immune responses, and other forms of cell death to provide more effective therapeutic interventions. Therefore, future therapeutic strategies for AMD should aim for a more holistic approach, targeting multiple pathways involved in retinal degeneration.

## 8. Conclusions

AMD entails gradual macular deterioration, notably affecting the RPE and photoreceptor. Persistent phagocytosis of photoreceptor outer segments over a lifetime leads to iron buildup and lipid peroxidation. Emerging research has illuminated the involvement of ferroptosis in AMD development. Iron accumulation in the retina, alongside oxidative stress and compromised antioxidant mechanisms, collectively exacerbate RPE dysfunction and photoreceptor degeneration. Extensive research has delved into the mechanistic underpinnings of ferroptosis and its potential implications in AMD. Diverse agents formulated as ferroptosis inhibitors have been meticulously crafted and synthesized to confer protective effects on retinal tissue. Nevertheless, AMD is a multifaceted disorder with various forms of cell death, such as apoptosis, pyroptosis, and necroptosis, which potentially contribute to retinal cell demise. It remains unclear whether multiple cell types in the retina undergo ferroptosis and how ferroptosis interacts with other forms of cell death crosstalk. Despite ferroptosis being the most recently unveiled form of cell death, its inhibitors may not entirely quell retinal cell death in AMD due to the complexity of backgrounds. Furthermore, oxidative stress is inexorably linked to multiple cell death pathways, and complicates research efforts aimed at delineating therapeutic efficacy solely from pathways other than ferroptosis. Moreover, it is imperative to recognize that lipid peroxides play a role in modulating immune responses, which are pivotal processes in AMD pathogenesis.

To effectively target ferroptosis in the context of AMD, several research directions are essential to further unravel the complex mechanisms involved. Firstly, a deeper investigation into the role of iron metabolism within RPE cells, photoreceptors, and other retinal cells will be crucial. Future studies should focus on how disruptions in iron uptake, storage, and export contribute to the ferroptosis in AMD. Cp and Heph can potentiate Fpn-mediated cellular iron export. An investigation uses the retina-specific Fpn KO mice to test the hypothesis that retinal iron overload in Cp/Heph DKO mice is caused by impaired iron export from neurons and glia. An increase in the level of retinal ferrous iron caused by the absence of these ferroxidases, followed by uptake into cells by ferrous iron importers, is most likely necessary [39,175]. Secondarily, identifying reliable biomarkers of ferroptosis in the retina and systemic circulation would enable early diagnosis, interventions, and evaluation of therapeutic effects. Through protein–protein interaction (PPI) network analysis, six hub ferroptosis-related genes (Jun, Stat3, Hmox1, Atf3, Hspa5 and Ripk1) are ultimately identified in a mice model of retinal degeneration induced by light damage. They also reveal some miRNAs, TFs, and several potential target drugs that may interact with the six HFRGs. The study provides novel potential biomarkers, therapeutic targets, and new insights into the ferroptosis landscape in retinal degenerative diseases [176]. Furthermore, the exploration of ferroptosis inhibition strategies is important for retinal degeneration diseases. Gene editing techniques such as CRISPR/Cas9 may directly regulate the expression of key ferroptosis-related genes. Comprehensive histology, imaging, and bulk RNA sequencing reveal that InP/ZnS QDs cause retinal degeneration. InP/ZnS QDs inhibit the expression of splicing factor prpf8, resulting in gpx4b mRNA unsplicing, which finally deplete glutathione and induce ferroptosis and mitophagy. Another study reveals that knockout prpf8 or gpx4b with a CRISPR/Cas9 system causes retinal damage. On the other hand, overexpression of prpf8 or gpx4b or supplementation of glutathione can ameliorate the retinal degenerative damage caused by InP/ZnS QDs [177]. Lesion site-targeted melanin-like nanoparticles, ConA-MelNPs, are designed as a novel ferroptosis inhibitor for retinal degenerative diseases. In a preclinical dAMD mouse model, a single intravitreal injection of ConA-MelNPs yielded significant responses in electroretinograms and visually driven optomotor responses in visually impaired mice. ConA-MelNPs alleviate severe mitochondrial damage caused by oxidative stress and protect RPE cells from ferroptosis induced by NaIO_3_ [178]. Utilizing nanocarriers to deliver ferroptosis inhibitors directly to retinal tissue could improve drug bioavailability and minimize systemic side effects. Moreover, investigating the crosstalk between ferroptosis and other pathophysiological mechanisms is essential. Exploring how ferroptosis interacts with oxidative stress, chronic inflammation, and immune responses in AMD could help identify new combinatory treatment strategies. Consequently, delving into ferroptosis undoubtedly represents a groundbreaking stride in both foundational and clinical AMD research, offering renewed optimism and avenues for enhanced therapeutic interventions for AMD patients.

## Figures and Tables

**Table 1:** Summary of ferroptosis inhibitors.

Category	Drugs	Experimental Objects	Mechanisms and Targets	Effect	References
Iron-chelating agent	Deferoxamine	ARPE-19 cells	Decreased iron level	Enhanced cell viability	[151]
	Deferiprone	Hepc KO mice/rd6 mice	Decreased iron levels, alleviating oxidative stress	Preserved photoreceptor and RPE cell	[152,153]
	Deferasirox	Rats	Reduced iron content and oxidative stress	Protected retinal neurons	[154]
	Zinc deferriamine	rd10 mice	Reduced iron content and lipid peroxidation	Preserved photoreceptors	[155]
	Salicylaldehyde isonicotinoyl hydrazine	ARPE-19 cells	Reduced iron levels and ROS levels	Enhanced cell viability	[156]
	VK-28/VAR10303/M30	rd10 mice	Downregulation of Tf, reduced the production of TNF-α and IL-1β	Preserved photoreceptors, improved visual behaviors	[157]
	KCa[FeIII(CN)6]	C57BL/6J mice	Reduced iron levels	Halted the degeneration of RPE cells and photoreceptors	[158]
	Fenofibrate	ARPE-19 cells	Reduced iron levels and inhibited ROS generation by downregulating the Wnt/β-catenin signaling pathway	Enhanced cell viability	[159]
	Ferrostatin-1	HRPEpiC cells/661w cells	Reduced lipid peroxidation by mediating the GSH-GPX4 pathway	Enhanced viability of primary HRPEpiC cells	[120,160,161]
Lipophilic antioxidant	liproxstatin-1	ARPE-19 cells	Reduced lipid peroxidation	Rescued cell viability	[13]
	Zileuton	ARPE-19 cells/mice	Inhibited 5-LOX	Increased cell viability, reduced photoreceptor death	[162]
	Astragaloside IV	ARPE-19 cells	Promoted the expression of Sirt1 and Nrf2	Decreased cell death	[163]
	L-carnitine	HRPE cells	Alleviated oxidative damage	Protected RPE cells	[164]
	Ommochromes	ARPE-19 cells	Mitigated the levels of inflammatory cytokines and oxidative stress	Inhibited ferroptosis	[165]

## References

[1] Pennington K.L., DeAngelis M.M. (2016). Epidemiology of age-related macular degeneration (AMD): Associations with cardiovascular disease phenotypes and lipid factors. Eye Vis..

[2] Ach T., Huisingh C., McGwin G., Messinger J.D., Zhang T., Bentley M.J., Gutierrez D.B., Ablonczy Z., Smith R.T., Sloan K.R. (2014). Quantitative autofluorescence and cell density maps of the human retinal pigment epithelium. Investig. Ophthalmol. Vis. Sci..

[3] Strauss O. (2005). The Retinal Pigment Epithelium in Visual Function. Physiol. Rev..

[4] Hanus J., Anderson C., Wang S. (2015). RPE necroptosis in response to oxidative stress and in AMD. Ageing Res. Rev..

[5] Datta S., Cano M., Ebrahimi K., Wang L., Handa J.T. (2017). The impact of oxidative stress and inflammation on RPE degeneration in non-neovascular AMD. Prog. Retin. Eye Res..

[6] Ding J.D., Johnson L.V., Herrmann R., Farsiu S., Smith S.G., Groelle M., Mace B.E., Sullivan P., Jamison J.A., Kelly U. (2011). Anti-amyloid therapy protects against retinal pigmented epithelium damage and vision loss in a model of age-related macular degeneration. Proc. Natl. Acad. Sci. USA.

[7] Ramkumar H.L., Zhang J., Chan C.C. (2010). Retinal ultrastructure of murine models of dry age-related macular degeneration (AMD). Prog. Retin. Eye Res..

[8] Hobbs S.D., Pierce K. (2022). Wet Age-Related Macular Degeneration (Wet AMD).

[9] Ueda-Consolvo T., Ozaki H., Nakamura T., Oiwake T., Hayashi A. (2019). The association between cone density and visual function in the macula of patients with retinitis pigmentosa. Graefe’s Arch. Clin. Exp. Ophthalmol..

[10] Nashine S. (2021). Potential Therapeutic Candidates for Age-Related Macular Degeneration (AMD). Cells.

[11] Bowes Rickman C., Farsiu S., Toth C.A., Klingeborn M. (2013). Dry age-related macular degeneration: Mechanisms, therapeutic targets, and imaging. Investig. Ophthalmol. Vis. Sci..

[12] Jonasson F., Arnarsson A., Eiríksdottir G., Harris T.B., Launer L.J., Meuer S.M., Klein B.E., Klein R., Gudnason V., Cotch M.F. (2011). Prevalence of age-related macular degeneration in old persons: Age, Gene/environment Susceptibility Reykjavik Study. Ophthalmology.

[13] Zhao X., Gao M., Liang J., Chen Y., Wang Y., Wang Y., Xiao Y., Zhao Z., Wan X., Jiang M. (2021). SLC7A11 Reduces Laser-Induced Choroidal Neovascularization by Inhibiting RPE Ferroptosis and VEGF Production. Front. Cell Dev. Biol..

[14] Dixon S.J., Lemberg K.M., Lamprecht M.R., Skouta R., Zaitsev E.M., Gleason C.E., Patel D.N., Bauer A.J., Cantley A.M., Yang W.S. (2012). Ferroptosis: An iron-dependent form of nonapoptotic cell death. Cell.

[15] Friedmann Angeli J.P., Schneider M., Proneth B., Tyurina Y.Y., Tyurin V.A., Hammond V.J., Herbach N., Aichler M., Walch A., Eggenhofer E. (2014). Inactivation of the ferroptosis regulator Gpx4 triggers acute renal failure in mice. Nat. Cell Biol..

[16] Jenkins N.L., James S.A., Salim A., Sumardy F., Speed T.P., Conrad M., Richardson D.R., Bush A.I., McColl G. (2020). Changes in ferrous iron and glutathione promote ferroptosis and frailty in aging Caenorhabditis elegans. eLife.

[17] Brock J.H. (1989). B Iron-binding proteins. Acta Paediatr. Scand. Suppl..

[18] Liu K., Li H., Wang F., Su Y. (2023). Ferroptosis: Mechanisms and advances in ocular diseases. Mol. Cell Biochem..

[19] Yant L.J., Ran Q., Rao L., Van Remmen H., Shibatani T., Belter J.G., Motta L., Richardson A., Prolla T.A. (2003). The selenoprotein GPX4 is essential for mouse development and protects from radiation and oxidative damage insults. Free Radic. Biol. Med..

[20] Rochette L., Dogon G., Rigal E., Zeller M., Cottin Y., Vergely C. (2023). Lipid Peroxidation and Iron Metabolism: Two Corner Stones in the Homeostasis Control of Ferroptosis. Int. J. Mol. Sci..

[21] Koppula P., Zhang Y., Zhuang L., Gan B. (2018). Amino acid transporter SLC7A11/xCT at the crossroads of regulating redox homeostasis and nutrient dependency of cancer. Cancer Commun..

[22] Tu H., Tang L.-J., Luo X.-J., Ai K.-L., Peng J. (2021). Insights into the novel function of system Xc- in regulated cell death. Eur. Rev. Med. Pharmacol. Sci..

[23] Sun Y., Zheng Y., Wang C., Liu Y. (2018). Glutathione depletion induces ferroptosis, autophagy, and premature cell senescence in retinal pigment epithelial cells. Cell Death Dis..

[24] Sreekumar P.G., Ferrington D.A., Kannan R. (2021). Glutathione Metabolism and the Novel Role of Mitochondrial GSH in Retinal Degeneration. Antioxidants.

[25] Deponte M. (2013). Glutathione catalysis and the reaction mechanisms of glutathione-dependent enzymes. Biochim. Biophys. Acta (BBA)-Gen. Subj..

[26] Ursini F., Maiorino M. (2020). Lipid peroxidation and ferroptosis: The role of GSH and GPx4. Free Radic. Biol. Med..

[27] Ingold I., Berndt C., Schmitt S., Doll S., Poschmann G., Buday K., Roveri A., Peng X., Freitas F.P., Seibt T. (2018). Selenium Utilization by GPX4 Is Required to Prevent Hydroperoxide-Induced Ferroptosis. Cell.

[28] Li F.-J., Long H.-Z., Zhou Z.-W., Luo H.-Y., Xu S.-G., Gao L.-C. (2022). System Xc−/GSH/GPX4 axis: An important antioxidant system for the ferroptosis in drug-resistant solid tumor therapy. Front. Pharmacol..

[29] Tonnus W., Gembardt F., Latk M., Parmentier S., Hugo C., Bornstein S.R., Linkermann A. (2018). The clinical relevance of necroinflammation—Highlighting the importance of acute kidney injury and the adrenal glands. Cell Death Differ..

[30] Liu M., Kong X.Y., Yao Y., Wang X.A., Yang W., Wu H., Li S., Ding J.W., Yang J. (2022). The critical role and molecular mechanisms of ferroptosis in antioxidant systems: A narrative review. Ann. Transl. Med..

[31] Wooff Y., Fernando N., Wong J.H.C., Dietrich C., Aggio-Bruce R., Chu-Tan J.A., Robertson A.A.B., Doyle S.L., Man S.M., Natoli R. (2020). Caspase-1-dependent inflammasomes mediate photoreceptor cell death in photo-oxidative damage-induced retinal degeneration. Sci. Rep..

[32] Kaarniranta K., Tokarz P., Koskela A., Paterno J., Blasiak J. (2017). Autophagy regulates death of retinal pigment epithelium cells in age-related macular degeneration. Cell Biol. Toxicol..

[33] Biesemeier A., Yoeruek E., Eibl O., Schraermeyer U. (2015). Iron accumulation in Bruch’s membrane and melanosomes of donor eyes with age-related macular degeneration. Exp. Eye Res..

[34] Ugarte M., Geraki K., Jeffery G. (2018). Aging results in iron accumulations in the non-human primate choroid of the eye without an associated increase in zinc, copper or sulphur. BioMetals.

[35] Jiang X., Stockwell B.R., Conrad M. (2021). Ferroptosis: Mechanisms, biology and role in disease. Nat. Rev. Mol. Cell Biol..

[36] Lüscher T.F. (2015). Ageing, inflammation, and oxidative stress: Final common pathways of cardiovascular disease. Eur. Heart J..

[37] Zhang S.-M., Fan B., Li Y.L., Zuo Z.-Y., Li G.-Y. (2023). Oxidative Stress-Involved Mitophagy of Retinal Pigment Epithelium and Retinal Degenerative Diseases. Cell. Mol. Neurobiol..

[38] Zou M., Ke Q., Nie Q., Qi R., Zhu X., Liu W., Hu X., Sun Q., Fu J.-L., Tang X. (2022). Inhibition of cGAS-STING by JQ1 alleviates oxidative stress-induced retina inflammation and degeneration. Cell Death Differ..

[39] Fleckenstein M., Keenan T.D., Guymer R.H., Chakravarthy U., Schmitz-Valckenberg S., Klaver C.C., Wong W.T., Chew E.Y. (2021). Age-related macular degeneration. Nat. Rev. Dis. Primers.

[40] Hyttinen J.M., Blasiak J., Kaarniranta K. (2023). Non-Coding RNAs Regulating Mitochondrial Functions and the Oxidative Stress Response as Putative Targets against Age-Related Macular Degeneration (AMD). Int. J. Mol. Sci..

[41] Yu Y., Yan Y., Niu F., Wang Y., Chen X., Su G., Liu Y., Zhao X., Qian L., Liu P. (2021). Ferroptosis: A cell death connecting oxidative stress, inflammation and cardiovascular diseases. Cell Death Discov..

[42] Lim L.S., Mitchell P., Seddon J.M., Holz F.G., Wong T.Y. (2012). Age-related macular degeneration. Lancet.

[43] Bhutto I., Lutty G. (2012). Understanding age-related macular degeneration (AMD): Relationships between the photoreceptor/retinal pigment epithelium/Bruch’s membrane/choriocapillaris complex. Mol. Asp. Med..

[44] Kokkinopoulos I., Shahabi G., Colman A., Jeffery G. (2011). Mature peripheral RPE cells have an intrinsic capacity to proliferate; a potential regulatory mechanism for age-related cell loss. PLoS ONE.

[45] Rashid A., Bhatia S.K., Mazzitello K.I., Chrenek M.A., Zhang Q., Boatright J.H., Grossniklaus H.E., Jiang Y., Nickerson J.M. (2016). RPE Cell and Sheet Properties in Normal and Diseased Eyes. Adv. Exp. Med. Biol..

[46] Massé A., Buhannic L. (2017). Comprendre la dégénérescence maculaire liée à l’âge. Actual. Pharm..

[47] Bonilha V.L., Bell B.A., Hu J., Milliner C., Pauer G.J., Hagstrom S.A., Radu R.A., Hollyfield J.G. (2020). Geographic Atrophy: Confocal Scanning Laser Ophthalmoscopy, Histology, and Inflammation in the Region of Expanding Lesions. Investig. Ophthalmol. Vis. Sci..

[48] Yang M., So K.F., Lam W.C., Lo A.C.Y. (2020). Novel Programmed Cell Death as Therapeutic Targets in Age-Related Macular Degeneration?. Int. J. Mol. Sci..

[49] Song D., Kanu L.N., Li Y., Kelly K.L., Bhuyan R.K., Aleman T., Morgan J.I.W., Dunaief J.L. (2016). AMD-like retinopathy associated with intravenous iron. Exp. Eye Res..

[50] Chua S.Y.L., Khawaja A.P., Dick A.D., Morgan J., Dhillon B., Lotery A.J., Strouthidis N.G., Reisman C., Peto T., Khaw P.T. (2020). Ambient Air Pollution Associations with Retinal Morphology in the UK Biobank. Investig. Ophthalmol. Vis. Sci..

[51] Moiseyev G., Takahashi Y., Chen Y., Gentleman S., Redmond T.M., Crouch R.K., Ma J.X. (2006). RPE65 is an iron (II)-dependent isomerohydrolase in the retinoid visual cycle. J. Biol. Chem..

[52] Gnana-Prakasam J.P., Martin P.M., Smith S.B., Ganapathy V. (2010). Expression and function of iron-regulatory proteins in retina. IUBMB Life.

[53] Gnana-Prakasam J.P., Ananth S., Prasad P.D., Zhang M., Atherton S.S., Martin P.M., Smith S.B., Ganapathy V. (2011). Expression and iron-dependent regulation of succinate receptor GPR91 in retinal pigment epithelium. Investig. Ophthalmol. Vis. Sci..

[54] Liu J., Kang R., Tang D. (2022). Signaling pathways and defense mechanisms of ferroptosis. FEBS J..

[55] Kruszewski M. (2003). Labile iron pool: The main determinant of cellular response to oxidative stress. Mutat. Res..

[56] Steere A.N., Byrne S.L., Chasteen N.D., Mason A.B. (2012). Kinetics of iron release from transferrin bound to the transferrin receptor at endosomal pH. Biochim. Biophys. Acta.

[57] Zhao T., Guo X., Sun Y. (2021). Iron Accumulation and Lipid Peroxidation in the Aging Retina: Implication of Ferroptosis in Age-Related Macular Degeneration. Aging Dis..

[58] Puri C. (2009). Loss of Myosin VI No Insert Isoform (NoI) Induces a Defect in Clathrin-mediated Endocytosis and Leads to Caveolar Endocytosis of Transferrin Receptor. J. Biol. Chem..

[59] Sterling J., Guttha S., Song Y., Song D., Hadziahmetovic M., Dunaief J.L. (2017). Iron importers Zip8 and Zip14 are expressed in retina and regulated by retinal iron levels. Exp. Eye Res..

[60] Shvartsman M., Ioav Cabantchik Z. (2012). Intracellular iron trafficking: Role of cytosolic ligands. BioMetals.

[61] Rice A.E., Mendez M.J., Hokanson C.A., Rees D.C., Bjorkman P.J. (2009). Investigation of the biophysical and cell biological properties of ferroportin, a multipass integral membrane protein iron exporter. J. Mol. Biol..

[62] Ashok A., Chaudhary S., Wise A.S., Rana N.A., McDonald D., Kritikos A.E., Lindner E., Singh N. (2021). Release of Iron-Loaded Ferritin in Sodium Iodate-Induced Model of Age Related Macular Degeneration: An In-Vitro and In-Vivo Study. Antioxidants.

[63] Chowers I., Wong R., Dentchev T., Farkas R.H., Iacovelli J., Gunatilaka T.L., Medeiros N.E., Presley J.B., Campochiaro P.A., Curcio C.A. (2006). The iron carrier transferrin is upregulated in retinas from patients with age-related macular degeneration. Investig. Ophthalmol. Vis. Sci..

[64] Yeo J.H., Colonne C.K., Tasneem N., Cosgriff M.P., Fraser S.T. (2019). The iron islands: Erythroblastic islands and iron metabolism. Biochim. Biophys. Acta Gen. Subj..

[65] Muckenthaler M.U., Rivella S., Hentze M.W., Galy B. (2017). A Red Carpet for Iron Metabolism. Cell.

[66] Kawabata H., Germain R.S., Vuong P.T., Nakamaki T., Said J.W., Koeffler H.P. (2000). Transferrin receptor 2-alpha supports cell growth both in iron-chelated cultured cells and in vivo. J. Biol. Chem..

[67] Lok C.N., Ponka P. (1999). Identification of a hypoxia response element in the transferrin receptor gene. J. Biol. Chem..

[68] Martin P.M., Gnana-Prakasam J.P., Roon P., Smith R.G., Smith S.B., Ganapathy V. (2006). Expression and polarized localization of the hemochromatosis gene product HFE in retinal pigment epithelium. Investig. Ophthalmol. Vis. Sci..

[69] Testi C., Boffi A., Montemiglio L.C. (2019). Structural analysis of the transferrin receptor multifaceted ligand(s) interface. Biophys. Chem..

[70] Wang Z., Ding Y., Wang X., Lu S., Wang C., He C., Wang L., Piao M., Chi G., Luo Y. (2018). Pseudolaric acid B triggers ferroptosis in glioma cells via activation of Nox4 and inhibition of xCT. Cancer Lett..

[71] Imamura T., Hirayama T., Tsuruma K., Shimazawa M., Nagasawa H., Hara H. (2014). Hydroxyl radicals cause fluctuation in intracellular ferrous ion levels upon light exposure during photoreceptor cell death. Exp. Eye Res..

[72] Kawabata H. (2019). Transferrin and transferrin receptors update. Free Radic. Biol. Med..

[73] Knovich M.A., Storey J.A., Coffman L.G., Torti S.V., Torti F.M. (2009). Ferritin for the clinician. Blood Rev..

[74] Brissot P., Ropert M., Le Lan C., Loreal O. (2012). Non-transferrin bound iron: A key role in iron overload and iron toxicity. Biochim. Biophys. Acta (BBA)-Gen. Subj..

[75] Tang D., Chen X., Kang R., Kroemer G. (2021). Ferroptosis: Molecular mechanisms and health implications. Cell Res..

[76] Ghosh M.C., Zhang D.L., Rouault T.A. (2015). Iron misregulation and neurodegenerative disease in mouse models that lack iron regulatory proteins. Neurobiol. Dis..

[77] Zhang D.L., Ghosh M.C., Rouault T.A. (2014). The physiological functions of iron regulatory proteins in iron homeostasis—An update. Front. Pharmacol..

[78] Hirota K. (2019). An intimate crosstalk between iron homeostasis and oxygen metabolism regulated by the hypoxia-inducible factors (HIFs). Free Radic. Biol. Med..

[79] Bourdon E., Kang D.K., Ghosh M.C., Drake S.K., Wey J., Levine R.L., Rouault T.A. (2003). The role of endogenous heme synthesis and degradation domain cysteines in cellular iron-dependent degradation of IRP2. Blood Cells Mol. Dis..

[80] Fuhrmann D.C., Mondorf A., Beifuß J., Jung M., Brüne B. (2020). Hypoxia inhibits ferritinophagy, increases mitochondrial ferritin, and protects from ferroptosis. Redox Biol..

[81] Cronin S.J.F., Woolf C.J., Weiss G., Penninger J.M. (2019). The Role of Iron Regulation in Immunometabolism and Immune-Related Disease. Front. Mol. Biosci..

[82] Zhang D.L., Wu J., Shah B.N., Greutélaers K.C., Ghosh M.C., Ollivierre H., Su X.Z., Thuma P.E., Bedu-Addo G., Mockenhaupt F.P. (2018). Erythrocytic ferroportin reduces intracellular iron accumulation, hemolysis, and malaria risk. Science.

[83] Gnana-Prakasam J.P., Martin P.M., Mysona B.A., Roon P., Smith S.B., Ganapathy V. (2008). Hepcidin expression in mouse retina and its regulation via lipopolysaccharide/Toll-like receptor-4 pathway independent of Hfe. Biochem. J..

[84] Darshan D., Anderson G.J. (2009). Interacting signals in the control of hepcidin expression. BioMetals.

[85] Aschemeyer S., Qiao B., Stefanova D., Valore E.V., Sek A.C., Ruwe T.A., Vieth K.R., Jung G., Casu C., Rivella S. (2018). Structure-function analysis of ferroportin defines the binding site and an alternative mechanism of action of hepcidin. Blood.

[86] Stockwell B.R., Friedmann Angeli J.P., Bayir H., Bush A.I., Conrad M., Dixon S.J., Fulda S., Gascón S., Hatzios S.K., Kagan V.E. (2017). Ferroptosis: A Regulated Cell Death Nexus Linking Metabolism, Redox Biology, and Disease. Cell.

[87] Bang S., Park S., Lee Y.M., Hong S., Cho K.B., Nam W. (2014). Demonstration of the heterolytic O-O bond cleavage of putative nonheme iron(II)-OOH(R) complexes for Fenton and enzymatic reactions. Angew. Chem. Int. Ed. Engl..

[88] Michalski M.C., Calzada C., Makino A., Michaud S., Guichardant M. (2008). Oxidation products of polyunsaturated fatty acids in infant formulas compared to human milk–A preliminary study. Mol. Nutr. Food Res..

[89] Masaldan S., Bush A.I., Devos D., Rolland A.S., Moreau C. (2019). Striking while the iron is hot: Iron metabolism and ferroptosis in neurodegeneration. Free Radic. Biol. Med..

[90] Dixon S.J., Winter G.E., Musavi L.S., Lee E.D., Snijder B., Rebsamen M., Superti-Furga G., Stockwell B.R. (2015). Human Haploid Cell Genetics Reveals Roles for Lipid Metabolism Genes in Nonapoptotic Cell Death. ACS Chem. Biol..

[91] Wong-Ekkabut J., Xu Z., Triampo W., Tang I.M., Tieleman D.P., Monticelli L. (2007). Effect of lipid peroxidation on the properties of lipid bilayers: A molecular dynamics study. Biophys. J..

[92] SanGiovanni J.P., Chew E.Y., Clemons T.E., Ferris F.L., Gensler G., Lindblad A.S., Milton R.C., Seddon J.M., Klein R., Sperduto R.D. (2007). Age-Related Eye Disease Study Research Group (2008 Sep). The relationship of dietary omega-3 long-chain polyunsaturated fatty acid intake with incident age-related macular degeneration: AREDS report no. 23. Arch. Ophthalmol..

[93] Hirschhorn T., Stockwell B.R. (2019). The development of the concept of ferroptosis. Free Radic. Biol. Med..

[94] Chen C., Chen J., Wang Y., Liu Z., Wu Y. (2021). Ferroptosis drives photoreceptor degeneration in mice with defects in all-trans-retinal clearance. J. Biol. Chem..

[95] Ng M.Y.W., Wai T., Simonsen A. (2021). Quality control of the mitochondrion. Dev. Cell.

[96] Losón O.C., Song Z., Chen H., Chan D.C. (2013). Fis1, Mff, MiD49, and MiD51 mediate Drp1 recruitment in mitochondrial fission. Mol. Biol. Cell.

[97] Romanello V., Sandri M. (2021). The connection between the dynamic remodeling of the mitochondrial network and the regulation of muscle mass. Cell. Mol. Life Sci..

[98] Mattie S., Riemer J., Wideman J.G., McBride H.M. (2017). A new mitofusin topology places the redox-regulated C terminus in the mitochondrial intermembrane space. J. Cell Biol..

[99] Feher J., Kovacs I., Artico M., Cavallotti C., Papale A., Gabrieli C.B. (2006). Mitochondrial alterations of retinal pigment epithelium in age-related macular degeneration. Neurobiol. Aging.

[100] Kaarniranta K., Uusitalo H., Blasiak J., Felszeghy S., Kannan R., Kauppinen A., Salminen A., Sinha D., Ferrington D. (2020). Mechanisms of mitochondrial dysfunction and their impact on age-related macular degeneration. Prog. Retin. Eye Res..

[101] Wang L., Yu X., Zhang D., Wen Y., Zhang L., Xia Y., Chen J., Xie C., Zhu H., Tong J. (2023). Long-term blue light exposure impairs mitochondrial dynamics in the retina in light-induced retinal degeneration in vivo and in vitro. J. Photochem. Photobiol. B Biol..

[102] Li J., Feng Y., Li Y., He P., Zhou Q., Tian Y., Yao R., Yao Y. (2024). Ferritinophagy: A novel insight into the double-edged sword in ferritinophagy–ferroptosis axis and human diseases. Cell Prolif..

[103] Moroishi T., Yamauchi T., Nishiyama M., Nakayama K.I. (2014). HERC2 Targets the Iron Regulator FBXL5 for Degradation and Modulates Iron Metabolism. J. Biol. Chem..

[104] Mancias J.D., Vaites L.P., Nissim S., Biancur E.D., Kim A.J., Wang X., Liu Y., Goessling W., Kimmelman A.C., Harper J.W. (2015). Ferritinophagy via NCOA4 is required for erythropoiesis and is regulated by iron dependent HERC2-mediated proteolysis. eLife.

[105] Yao F., Peng J., Zhang E., Ji D., Gao Z., Tang Y., Yao X., Xia X. (2023). Pathologically high intraocular pressure disturbs normal iron homeostasis and leads to retinal ganglion cell ferroptosis in glaucoma. Cell Death Differ..

[106] Li H.Y., Wei T.T., Zhuang M., Tan C.Y., Xie T.H., Cai J., Yao Y., Zhu L. (2023). Iron derived from NCOA4-mediated ferritinophagy causes cellular senescence via the cGAS-STING pathway. Cell Death Discov..

[107] Stoyanovsky D.A., Tyurina Y.Y., Shrivastava I., Bahar I., Tyurin V.A., Protchenko O., Jadhav S., Bolevich S.B., Kozlov A.V., Vladimirov Y.A. (2019). Iron catalysis of lipid peroxidation in ferroptosis: Regulated enzymatic or random free radical reaction?. Free Radic. Biol. Med..

[108] Hentze M.W., Muckenthaler M.U., Galy B., Camaschella C. (2010). Two to Tango: Regulation of Mammalian Iron Metabolism. Cell.

[109] Torti S.V., Torti F.M. (2013). Iron and cancer: More ore to be mined. Nat. Rev. Cancer.

[110] Xiang Y., Song X., Long D. (2024). Ferroptosis regulation through Nrf2 and implications for neurodegenerative diseases. Arch. Toxicol..

[111] Imai H., Matsuoka M., Kumagai T., Sakamoto T., Koumura T. (2016). Lipid Peroxidation-Dependent Cell Death Regulated by GPx4 and Ferroptosis. Apoptotic and Non-Apoptotic Cell Death.

[112] Conrad M., Proneth B. (2020). Selenium: Tracing Another Essential Element of Ferroptotic Cell Death. Cell Chem. Biol..

[113] Shimada K., Hayano M., Pagano N.C., Stockwell B.R. (2016). Cell-Line Selectivity Improves the Predictive Power of Pharmacogenomic Analyses and Helps Identify NADPH as Biomarker for Ferroptosis Sensitivity. Cell Chem. Biol..

[114] Forcina G.C., Dixon S.J. (2019). GPX4 at the Crossroads of Lipid Homeostasis and Ferroptosis. Proteomics.

[115] Koppula P., Zhuang L., Gan B. (2021). Cystine transporter SLC7A11/xCT in cancer: Ferroptosis, nutrient dependency, and cancer therapy. Protein Cell.

[116] Jiang L., Kon N., Li T., Wang S.J., Su T., Hibshoosh H., Baer R., Gu W. (2015). Ferroptosis as a p53-mediated activity during tumour suppression. Nature.

[117] Chen D., Tavana O., Chu B., Erber L., Chen Y., Baer R., Gu W. (2017). NRF2 Is a Major Target of ARF in p53-Independent Tumor Suppression. Mol. Cell.

[118] Zhang Y., Shi J., Liu X., Feng L., Gong Z., Koppula P., Sirohi K., Li X., Wei Y., Lee H. (2018). BAP1 links metabolic regulation of ferroptosis to tumour suppression. Nat. Cell Biol..

[119] Tang W., Guo J., Liu W., Ma J., Xu G. (2021). Ferrostatin-1 attenuates ferroptosis and protects the retina against light-induced retinal degeneration. Biochem. Biophys. Res. Commun..

[120] Yang M., Tsui M.G., Tsang J.K.W., Goit R.K., Yao K.M., So K.F., Lam W.C., Lo A.C.Y. (2022). Involvement of FSP1-CoQ (10)-NADH and GSH-GPx-4 pathways in retinal pigment epithelium ferroptosis. Cell Death Dis..

[121] Xiang W., Li L., Zhao Q., Zeng Y., Shi J., Chen Z., Gao G., Lai K. (2023). PEDF protects retinal pigment epithelium from ferroptosis and ameliorates dry AMD-like pathology in a murine model. GeroScience.

[122] Yang Y., Wang Y., Deng Y., Lu J., Xiao L., Li J., Zhou Y., Nie F., Chen X., Peng J. (2023). Fructus Lycii and Salvia miltiorrhiza Bunge extract attenuate oxidative stress-induced photoreceptor ferroptosis in retinitis pigmentosa. Biomed. Pharmacother..

[123] Sakai O., Uchida T., Roggia M.F., Imai H., Ueta T., Amano S. (2015). Role of Glutathione Peroxidase 4 in Glutamate-Induced Oxytosis in the Retina. PLoS ONE.

[124] Xu J., Wu Y., Song P., Zhang M., Wang S., Zou M.-H. (2007). Proteasome-Dependent Degradation of Guanosine 5′-Triphosphate Cyclohydrolase I Causes Tetrahydrobiopterin Deficiency in Diabetes Mellitus. Circulation.

[125] Hu Q., Wei W., Wu D., Huang F., Li M., Li W., Yin J., Peng Y., Lu Y., Zhao Q. (2022). Blockade of GCH1/BH4 Axis Activates Ferritinophagy to Mitigate the Resistance of Colorectal Cancer to Erastin-Induced Ferroptosis. Front. Cell Dev. Biol..

[126] Costigan M., Latremoliere A., Woolf C.J. (2012). Analgesia by inhibiting tetrahydrobiopterin synthesis. Curr. Opin. Pharmacol..

[127] Kraft V.A.N., Bezjian C.T., Pfeiffer S., Ringelstetter L., Müller C., Zandkarimi F., Merl-Pham J., Bao X., Anastasov N., Kössl J. (2019). GTP Cyclohydrolase 1/Tetrahydrobiopterin Counteract Ferroptosis through Lipid Remodeling. ACS Central Sci..

[128] Cronin S.J., Yu W., Hale A., Licht-Mayer S., Crabtree M.J., Korecka J.A., Tretiakov E.O., Sealey-Cardona M., Somlyay M., Onji M. (2023). Crucial neuroprotective roles of the metabolite BH4 in dopaminergic neurons. bioRxiv.

[129] Bersuker K., Hendricks J.M., Li Z., Magtanong L., Ford B., Tang P.H., Roberts M.A., Tong B., Maimone T.J., Zoncu R. (2019). The CoQ oxidoreductase FSP1 acts parallel to GPX4 to inhibit ferroptosis. Nature.

[130] Yan H.F., Zou T., Tuo Q.Z., Xu S., Li H., Belaidi A.A., Lei P. (2021). Ferroptosis: Mechanisms and links with diseases. Signal Transduct. Target. Ther..

[131] Lee J., Roh J.L. (2023). Unleashing Ferroptosis in Human Cancers: Targeting Ferroptosis Suppressor Protein 1 for Overcoming Therapy Resistance. Antioxidants.

[132] Shukla S., Dubey K.K. (2018). CoQ10 a super-vitamin: Review on application and biosynthesis. 3 Biotech.

[133] Song Y., Lei H., Yu D., Zhu H., Hao M., Cui R., Meng X., Sheng X., Zhang L. (2023). Endogenous chemicals guard health through inhibiting ferroptotic cell death. BioFactors.

[134] Doll S., Freitas F.P., Shah R., Aldrovandi M., da Silva M.C., Ingold I., Goya Grocin A., Xavier da Silva T.N., Panzilius E., Scheel C.H. (2019). FSP1 is a glutathione-independent ferroptosis suppressor. Nature.

[135] Alehagen U., Johansson P., Aaseth J., Alexander J., Brismar K. (2017). Increase in insulin-like growth factor 1 (IGF-1) and insulin-like growth factor binding protein 1 after supplementation with selenium and coenzyme Q10. A prospective randomized double-blind placebo-controlled trial among elderly Swedish citizens. PLoS ONE.

[136] Miriyala S., Thippakorn C., Chaiswing L., Xu Y., Noel T., Tovmasyan A., Batinic-Haberle I., Kooi C.W.V., Chi W., Latif A.A. (2016). Novel role of 4-hydroxy-2-nonenal in AIFm2-mediated mitochondrial stress signaling. Free Radic. Biol. Med..

[137] Yin S., Kabashima T., Zhu Q., Shibata T., Kai M. (2017). Fluorescence assay of dihydroorotate dehydrogenase that may become a cancer biomarker. Sci. Rep..

[138] Zhou Y., Tao L., Zhou X., Zuo Z., Gong J., Liu X., Zhou Y., Liu C., Sang N., Liu H. (2021). DHODH and cancer: Promising prospects to be explored. Cancer Metab..

[139] Zhang J., Zhu Q., Peng Z., Li X.J., Ding P.F., Gao S., Sheng B., Liu Y., Lu Y., Zhuang Z. (2024). Menaquinone-4 attenuates ferroptosis by upregulating DHODH through activation of SIRT1 after subarachnoid hemorrhage. Free Radic. Biol. Med..

[140] Dodson M., de la Vega M.R., Cholanians A.B., Schmidlin C.J., Chapman E., Zhang D.D. (2019). Modulating NRF2 in Disease: Timing Is Everything. Annu. Rev. Pharmacol. Toxicol..

[141] Hellyer J.A., Padda S.K., Diehn M., Wakelee H.A. (2021). Clinical Implications of KEAP1-NFE2L2 Mutations in NSCLC. J. Thorac. Oncol..

[142] Tonelli C., Chio I.I.C., Tuveson D.A. (2018). Transcriptional Regulation by Nrf2. Antioxid. Redox Signal..

[143] Dodson M., Castro-Portuguez R., Zhang D.D. (2019). NRF2 plays a critical role in mitigating lipid peroxidation and ferroptosis. Redox Biol..

[144] Cho H.Y., Gladwell W., Wang X., Chorley B., Bell D., Reddy S.P., Kleeberger S.R. (2010). Nrf2-regulated PPARγ expression is critical to protection against acute lung injury in mice. Am. J. Respir. Crit. Care Med..

[145] Hayes J.D., Dinkova-Kostova A.T. (2014). The Nrf2 regulatory network provides an interface between redox and intermediary metabolism. Trends Biochem. Sci..

[146] Song Q., Jian W., Zhang Y., Li Q., Zhao Y., Liu R., Zeng Y., Zhang F., Duan J. (2024). Puerarin Attenuates Iron Overload—Induced Ferroptosis in Retina through a Nrf2—Mediated Mechanism. Mol. Nutr. Food Res..

[147] Li Y., Zhu X., Wang K., Zhu L., Murray M., Zhou F. (2022). *Ginkgo biloba* extracts (GBE) protect human RPE cells from *t-BHP*-induced oxidative stress and necrosis by activating the Nrf2-mediated antioxidant defence. J. Pharm. Pharmacol..

[148] Hybertson B., Gao B., Doan A. (2014). The clinical potential of influencing Nrf2 signaling in degenerative and immunological disorders. Clin. Pharmacol. Adv. Appl..

[149] Wang L., Ebrahimi K.B., Chyn M., Cano M., Handa J.T. (2016). Biology of p62/sequestosome-1 in Age-Related Macular Degeneration (AMD). Adv. Exp. Med. Biol..

[150] Zhao Z., Chen Y., Wang J., Sternberg P., Freeman M.L., Grossniklaus H.E., Cai J. (2011). Age-Related Retinopathy in NRF2-Deficient Mice. PLoS ONE.

[151] Totsuka K., Ueta T., Uchida T., Roggia M.F., Nakagawa S., Vavvas D.G., Honjo M., Aihara M. (2019). Oxidative stress induces ferroptotic cell death in retinal pigment epithelial cells. Exp. Eye Res..

[152] Song D., Zhao L., Li Y., Hadziahmetovic M., Song Y., Connelly J., Spino M., Dunaief J.L. (2014). The Oral Iron Chelator Deferiprone Protects Against Systemic Iron Overload–Induced Retinal Degeneration in Hepcidin Knockout Mice. Investig. Opthalmology Vis. Sci..

[153] Hadziahmetovic M., Pajic M., Grieco S., Song Y., Song D., Li Y., Cwanger A., Iacovelli J., Chu S., Ying G.-S. (2012). The Oral Iron Chelator Deferiprone Protects Against Retinal Degeneration Induced through Diverse Mechanisms. Transl. Vis. Sci. Technol..

[154] Sakamoto K., Suzuki T., Takahashi K., Koguchi T., Hirayama T., Mori A., Nakahara T., Nagasawa H., Ishii K. (2018). Iron-chelating agents attenuate NMDA-Induced neuronal injury via reduction of oxidative stress in the rat retina. Exp. Eye Res..

[155] Obolensky A., Berenshtein E., Lederman M., Bulvik B., Alper-Pinus R., Yaul R., Deleon E., Chowers I., Chevion M., Banin E. (2011). Zinc–desferrioxamine attenuates retinal degeneration in the rd10 mouse model of retinitis pigmentosa. Free Radic. Biol. Med..

[156] Lukinova N., Iacovelli J., Dentchev T., Wolkow N., Hunter A., Amado D., Ying G.-S., Sparrow J.R., Dunaief J.L. (2009). Iron chelation protects the retinal pigment epithelial cell line ARPE-19 against cell death triggered by diverse stimuli. Investig. Ophthalmol. Vis. Sci..

[157] Lin B., Youdim M.B.H. (2021). The protective, rescue and therapeutic potential of multi-target iron-chelators for retinitis pigmentosa. Free Radic. Biol. Med..

[158] Tang Z., Huo M., Ju Y., Dai X., Ni N., Liu Y., Gao H., Zhang D., Sun H., Fan X. (2021). Nanoprotection Against Retinal Pigment Epithelium Degeneration via Ferroptosis Inhibition. Small Methods.

[159] Mandala A., Armstrong A., Girresch B., Zhu J., Chilakala A., Chavalmane S., Chaudhary K., Biswas P., Ogilvie J., Gnana-Prakasam J.P. (2020). Fenofibrate prevents iron induced activation of canonical Wnt/β-catenin and oxidative stress signaling in the retina. npj Aging Mech. Dis..

[160] Liu B., Wang W., Shah A., Yu M., Liu Y., He L., Dang J., Yang L., Yan M., Ying Y. (2021). Sodium iodate induces ferroptosis in human retinal pigment epithelium ARPE-19 cells. Cell Death Dis..

[161] Gao S., Gao S., Wang Y., Li N., Yang Z., Yao H., Chen Y., Cheng Y., Zhong Y., Shen X. (2023). Inhibition of Ferroptosis Ameliorates Photoreceptor Degeneration in Experimental Diabetic Mice. Int. J. Mol. Sci..

[162] Lee J.J., Chang-Chien G.P., Lin S., Hsiao Y.T., Ke M.C., Chen A., Lin T.K. (2022). 5-Lipoxygenase Inhibition Protects Retinal Pigment Epithelium from Sodium Iodate-Induced Ferroptosis and Prevents Retinal Degeneration. Oxid. Med. Cell. Longev..

[163] Tang X., Li X., Zhang D., Han W. (2022). Astragaloside-IV alleviates high glucose-induced ferroptosis in retinal pigment epithelial cells by disrupting the expression of miR-138-5p/Sirt1/Nrf2. Bioengineered.

[164] Shamsi F.A., Chaudhry I.A., Boulton M.E., Al-Rajhi A.A. (2009). L-Carnitine Protects Human Retinal Pigment Epithelial Cells from Oxidative Damage. Curr. Eye Res..

[165] Figon F., Casas J. (2019). Ommochromes in invertebrates: Biochemistry and cell biology. Biol. Rev. Camb. Philos. Soc..

[166] Brittenham G.M. (2011). Iron-chelating therapy for transfusional iron overload. N. Engl. J. Med..

[167] Li X., Zhu S., Qi F. (2023). Blue light pollution causes retinal damage and degeneration by inducing ferroptosis. J. Photochem. Photobiol. B..

[168] Timoshnikov V.A., Kobzeva T.V., Polyakov N.E., Kontoghiorghes G.J. (2020). Redox Interactions of Vitamin C and Iron: Inhibition of the Pro-Oxidant Activity by Deferiprone. Int. J. Mol. Sci..

[169] Hadziahmetovic M., Song Y., Wolkow N., Iacovelli J., Grieco S., Lee J., Lyubarsky A., Pratico D., Connelly J., Spino M. (2011). The Oral Iron Chelator Deferiprone Protects against Iron Overload–Induced Retinal Degeneration. Investig. Opthalmology Vis. Sci..

[170] Song D., Song Y., Hadziahmetovic M., Zhong Y., Dunaief J.L. (2012). Systemic administration of the iron chelator deferiprone protects against light-induced photoreceptor degeneration in the mouse retina. Free Radic. Biol. Med..

[171] Caro A.A., Commissariat A., Dunn C., Kim H., García S.L., Smith A., Strang H., Stuppy J., Desrochers L.P., Goodwin T.E. (2015). Prooxidant and antioxidant properties of salicylaldehyde isonicotinoyl hydrazone iron chelators in HepG2 cells. Biochim. Biophys. Acta (BBA)-Gen. Subj..

[172] Liu Y., Wang W., Li Y., Xiao Y., Cheng J., Jia J. (2015). The 5-Lipoxygenase Inhibitor Zileuton Confers Neuroprotection against Glutamate Oxidative Damage by Inhibiting Ferroptosis. Biol. Pharm. Bull..

[173] Ribas G.S., Vargas C.R., Wajner M. (2014). L-carnitine supplementation as a potential antioxidant therapy for inherited neurometabolic disorders. Gene.

[174] Lewis L.L.M., Dörschmann P., Seeba C., Thalenhorst T., Roider J., Iloki Assanga S.B., Ruiz J.C.G., Del Castillo Castro T., Rosas-Burgos E.C., Plascencia-Jatomea M. (2022). Properties of Cephalopod Skin Ommochromes to Inhibit Free Radicals, and the Maillard Reaction and Retino-Protective Mechanisms in Cellular Models Concerning Oxidative Stress, Angiogenesis, and Inflammation. Antioxidants.

[175] Liu Y., Baumann B., Song Y., Zhang K., Sterling J.K., Lakhal-Littleton S., Kozmik Z., Su G., Dunaief J.L. (2022). Minimal effect of conditional ferroportin KO in the neural retina implicates ferrous iron in retinal iron overload and degeneration. Exp. Eye Res..

[176] Lei X.-L., Yang Q.-L., Wei Y.-Z., Qiu X., Zeng H.-Y., Yan A.-M., Peng K., Li Y.-L., Rao F.-Q., Chen F.-H. (2023). Identification of a novel ferroptosis-related gene signature associated with retinal degeneration induced by light damage in mice. Heliyon.

[177] Zheng N., Liao T., Zhang C., Zhang Z., Yan S., Xi X., Ruan F., Yang C., Zhao Q., Deng W. (2024). Quantum Dots-caused Retinal Degeneration in Zebrafish Regulated by Ferroptosis and Mitophagy in Retinal Pigment Epithelial Cells through Inhibiting Spliceosome. Adv. Sci..

[178] Huang K., Deng H., Wang S., Zhang F., Huang G., Wang L., Liu J., Zhao X., Ren H., Yang G. (2024). Melanin-Like Nanomedicine Functions as a Novel RPE Ferroptosis Inhibitor to Ameliorate Retinal Degeneration and Visual Impairment in Dry Age-Related Macular Degeneration. Adv. Healthc. Mater..

